# Distinct microbial nitrogen cycling processes in the deepest part of the ocean

**DOI:** 10.1128/msystems.00243-24

**Published:** 2024-06-28

**Authors:** Yuhan Huang, Xinxu Zhang, Yu Xin, Jiwei Tian, Meng Li

**Affiliations:** 1 Archaeal Biology Center, Shenzhen Key Laboratory of Marine Microbiome Engineering, Institute for Advanced Study, Shenzhen University, Shenzhen, China; 2 Synthetic Biology Research Center, Institute for Advanced Study, Shenzhen University, Shenzhen, China; 3 College of Life Sciences and Oceanography, Shenzhen University, Shenzhen, China; 4 Key Laboratory of Marine Chemistry Theory and Technology, Ministry of Education, Institute for Advanced Ocean Study, Ocean University of China, Qingdao, Shandong, China; 5 MOE Key Laboratory of Physical Oceanography, Frontiers Science Center for Deep Ocean Multispheres and Earth System, Ocean University of China, Qingdao, China; Institute of Soil Science, Chinese Academy of Sciences, Nanjing, China; China University of Geosciences, Wuhan, Hubei, China

**Keywords:** anammox, deep biosphere, hadal trench, metagenomics, nitrite oxidation

## Abstract

The Mariana Trench (MT) is the deepest part of the ocean on Earth. Previous studies have described the microbial community structures and functional potential in the seawater and surface sediment of MT. Still, the metabolic features and adaptation strategies of the microorganisms involved in nitrogen cycling processes are poorly understood. In this study, comparative metagenomic approaches were used to study microbial nitrogen cycling in three MT habitats, including hadal seawater [9,600–10,500 m below sea level (mbsl)], surface sediments [0–46 cm below seafloor (cmbsf) at a water depth between 7,143 and 8,638 mbsl], and deep sediments (200–306 cmbsf at a water depth of 8,300 mbsl). We identified five new nitrite-oxidizing bacteria (NOB) lineages that had adapted to the oligotrophic MT slope sediment, *via* their CO_2_ fixation capability through the reductive tricarboxylic acid (rTCA) or Calvin-Benson-Bassham (CBB) cycle; an anammox bacterium might perform aerobic respiration and utilize sedimentary carbohydrates for energy generation because it contains genes encoding type A cytochrome *c* oxidase and complete glycolysis pathway. In seawater, abundant alkane-oxidizing *Ketobacter* species can fix inert N_2_ released from other denitrifying and/or anammox bacteria. This study further expands our understanding of microbial life in the largely unexplored deepest part of the ocean.

**IMPORTANCE:**

The metabolic features and adaptation strategies of the nitrogen cycling microorganisms in the deepest part of the ocean are largely unknown. This study revealed that anammox bacteria might perform aerobic respiration in response to nutrient limitation or O_2_ fluctuations in the Mariana Trench sediments. Meanwhile, an abundant alkane-oxidizing *Ketobacter* species could fix N_2_ in hadal seawater. This study provides new insights into the roles of hadal microorganisms in global nitrogen biogeochemical cycles. It substantially expands our understanding of the microbial life in the largely unexplored deepest part of the ocean.

## INTRODUCTION

The hadal biosphere refers to a trench area with a water depth greater than 6,000 m that accounts for 45% of the vertical depth of the ocean. This habitat is characterized by high hydrostatic pressure (>60 MPa), low temperature (<4°C), and relatively isolated environments (1–3). In general, the topography of a trench is shaped like a funnel consisting of slope and axis area. Importantly, the high rates of carbon turnover were measured in the sediments, which contributes to the accumulation of organic carbon and nitrogen compounds (e.g., nitrate and amino acids) (4–6). However, compared with the trench axis, the trench slope is steeper with a more complex topography (e.g., horst, graben, seamount, and ridges) and covers a much wider area (7, 8). Sinking organic material unevenly distributed on the slope can also slide from the top to the trench bottom *via* ocean crust movements (such as earthquakes) and seawater circulation. Therefore, the sediment layer of the slope is generally thinner than that at the trench bottom and thus has a lower content of organics and microorganisms (4, 6, 9).

Previous studies have shown that various bacterial and archaeal communities were detected in the MT. For example, *Proteobacteria*, *Bacteroidota*, *Marinisomatota,* and *Thaumarchaeota* were dominant groups in the hadal seawater (10). The common microorganisms in the surface sediment were *Proteobacteria*, *Chloroflexota*, *Planctomycetes*, *Thaumarchaeota,* and *Nanoarchaeota* (9, 11). Recently, the metabolic features and adaptation strategies of the hadal microbiome have been uncovered using multiple techniques, such as genomics (metagenomics and metatranscriptomics), *in situ* measurements, and laboratory incubations. For example, *Thaumarchaeota* in MT seawater possessed two distinct sets of adenosine-triphosphate (ATP) synthases (binding Na^+^ or H^+^) in response to the extreme hadal environment (12). *Chloroflexi* in the sediments of MT can utilize a wide range of organics, and these bacteria respond to poor nutrition and a periodically fluctuating environment with a “feast-or-famine” metabolic strategy (13). *Labrenzia aggregate* strains isolated from the MT (0–9,600 m) had increased the plasmidic gene numbers along with isolation depth and obtained the extra genetic potential mainly through plasmid exchange to resist the extremely high hydrostatic pressure (14). Dermacozines, a new secondary metabolite produced by the bacterial strain *Dermacoccus abyssi* MT1.1^T^ (isolated from MT sediment at 10,898 m), has multiple activities such as scavenging free radicals and cytotoxicity (15). Some *Alphaproteobacteria* isolates (e.g., *Sagittula stellata*, *Labrenzia aggregate*, *Pelagibaca bermudensis*) produce dimethylsulfoniopropionate, which protects bacteria against high hydrostatic pressure in the aphotic deep ocean (16). Taken together, adaptating microbial communities to the hadal environment may contribute to their metabolic activities and important roles in the global carbon and nitrogen biogeochemical cycles.

Nitrogen is a fundamental nutrient element in the environment. The microbial nitrogen cycle is a basic metabolic process to synthesize biological molecules (e.g., amino acids, proteins, and nucleic acids) during microbial growth and reproduction. However, bioavailable nitrogen (e.g., nitrate and ammonia) is scarce on Earth, and the microbial-mediated nitrogen cycling processes contribute to a significant fraction of bioavailable nitrogen in many environments (17, 18). In general, the nitrogen compounds in hadal environments consist of NO_3_^−^, NO_2_^−^, NH_4_^+^, and proteins. NH_4_^+^ can be released through microbial processes such as deamination and protein degradation, which are catalyzed by ammonia lyases, deaminases, and proteases (19). Constant concentrations of NO_3_^−^ (34–36 μM) and NH_4_^+^ (<1 µM) were observed throughout the hadal water of MT (10, 20). However, concentrations of these nitrogen compounds varied with depth in the sediment of MT. Relatively high concentrations of NO_3_^−^ (20–36 μM) and low concentrations of NO_2_^−^ (~0.1 µM) and NH_4_^+^ (0–5 μM) were detected in the surface sediment (<50 cmbsf) (6), but these nitrogen compounds showed the opposite trends in the deep sediment (21). Few studies have reported microbial nitrogen metabolisms in the MT surface sediment and seawater. For example, multiple microbial groups (e.g., *Proteobacteria*, *Planctomycetota,* and *Bacteroidota*) participate in NO_3_^−^ reduction [dissimilatory nitrate reduction to ammonium (DNRA) and denitrification] (6, 22, 23). In the surface sediment of the Atacama Trench (5–10 cm at 8,085 m) and Kermadec Trench (15–20 cm at 10,010 m), approximately 67% and 91% of N_2_ were derived from the anammox process (24). Furthermore, some anammox bacterial MAGs (metagenome-assembled genomes) were retrieved from the MT surface sediments (18–21 cm at 10,840 m) (6). The nitrogen fixation gene *nifH* was detected in MT seawater (>9,600 m) (25). For nitrification, ammonia-oxidizing archaea (AOA) dominated by *Thaumarchaeota* in hadal trenches perform autotrophic carbon fixation through ammonium oxidation (12). However, the metabolic features and adaption strategies of these anammox bacteria, other types of nitrifying bacteria (e.g., NOB), and N_2_ fixers have not yet been revealed in the hadal biosphere.

To fill these gaps in our knowledge, we have used comparative metagenomic approaches to reveal the unique features of the microbial nitrogen cycling processes in three different hadal habitats, including hadal seawater (9,600–10,500 mbsl), surface sediment (0–46 cmbsf at a water depth between 7,143 and 8,638 mbsl), and deep sediment (200–306 cmbsf at a water depth of 8,300 mbsl) (Table S1).

## MATERIALS AND METHODS

### Sample collection, DNA extraction, and metagenome sequencing

The nine deep sediment samples were collected from the MT slope in the western Pacific Ocean during a cruise in 2019 using the research vessel *Haida* (11.13° N, 142.33° E). The water depth was 8,300 mbsl (Fig. S1). One sediment core was collected by gravity corer, and the subsamples of 200, 213, 229, 242, 250, 260, 272, 290, and 306 cmbsf were used in this study. The samples were stored at −80°C immediately after recovery onboard. DNA was extracted from 0.5 g of each sediment using a DNeasy PowerSoil Pro Kit (Qiagen, Germany) according to the manufacturer’s instructions, and only the interior sections of the sediment were subsampled with a flame-sterilized scoop for DNA extraction. In parallel, blank controls for all sampling and DNA extractions were prepared using Milli-Q water (18.2 MΩ; Millipore, USA) filtered through the 0.22-µm mesh membrane. Metagenomic sequencing was performed on the NovaSeq 6000 platform (Illumina, USA) using 2 × 150 bp paired-end technology.

The six surface sediment samples were obtained from the MT slope at water depths between 7,143 and 8,638 mbsl as described elsewhere (6). All intact core samples were immediately subsampled onboard and frozen at −80°C until use. DNA was extracted from 10 g sediment of each sample using PowerMax Soil DNA Isolation Kit (MoBio, Germany) according to the manufacturer’s protocol. Sequencing was performed on NovaSeq 6000 platform (2 × 150 bp for S.7143.2 and S.7143.16) or Miseq platform (2  ×  300 bp for S.7850.0, S.8638.0, S.8638.36, and S.8638.44) (Illumina, USA).

The three seawater samples were collected from the MT Challenger Deep at water depths between 9,600 and 10,500 mbsl as described elsewhere (26). About 50L of each seawater was filtered onto a 0.22-µm polycarbonate membrane (GTTP, 142  mm, Millipore, USA). DNA was extracted using the SDS method as described previously (26). Sequencing was performed on the Illumina HiSeq X-Ten platform using 2 × 150 bp paired-end technology.

### Metagenomic assembly and binning

All the metagenomes used in this study were assembled and binned using the same method. Briefly, the raw metagenomic reads were trimmed using Trimmomatic (v.0.38) (27) with default parameters. All clean reads from the six surface sediments were pooled together before *de novo* assembly to one coassembly. Meanwhile, all clean reads from the three seawater samples were pooled together before *de novo* assembly to one coassembly. The clean reads were then assembled into contigs using SPAdes (v.3.15.0) (28) with the parameters: --meta -k 21,29,39,59,79,99. Metagenomic binning and quality assessment were performed as described elsewhere (29). Briefly, the binning was performed using MetaBAT 2 (v.2.12.1) (30), and optimized with DAS Tool (v.1.1) (31). The completeness, contamination, and heterogeneity of the recovered MAGs were determined based on lineage-specific conserved marker gene sets in each genome by CheckM (v.1.0.7) (32). In total, 498 MAGs with >50% completeness and <10% contamination were selected for downstream analysis as described elsewhere (6, 13, 22).

### Taxonomic classification and relative abundance

Taxonomic assignments of the MAGs were performed using the “classify_wf” workflow in the GTDB-Tk software (v.2.1.0) (33). The recovered MAGs were dereplicated at 95% identity with CoverM software (v.0.6.1) (https://github.com/wwood/CoverM) to avoid arbitrary mapping among highly similar genomes, and the relative abundance of the dereplicated MAGs in each metagenome was calculated using CoverM with the program “coverm genome.” The total microbial community composition of each metagenome was performed using Metaxa2 (v.2.1.3) (34) based on the identified 16S rRNA gene reads. The RPKM value {RPKM = Mapped reads/[Gene length (Kb) ×Total reads (million)]} was used to indicate the abundance of each nitrogen cycling gene, which was normalized to account for variations in gene length and data set size (3). Statistical significance of the relative abundance between the two samples was analyzed using the Mann-Whitney U test in SPSS (v.22.0), and differences were considered significant when *P* < 0.05 as described elsewhere (35, 36).

### Retrieval of anammox, NOB, and *Ketobacter* reference genomes

The collection of anammox, NOB, and *Ketobacter* reference genomes were obtained from three sources, which included the MAGs recovered from the metagenomes of this study, the published genomes downloaded from the NCBI GenBank database (37) (August 16th, 2022), and those from the genomic catalog of Earth’s microbiomes (38).

### Gene annotation and metabolic pathway reconstruction

The protein-coding genes were predicted by Prodigal (v.2.6.3) (39) using “-p meta” option. Orthologous gene families were identified by OrthoFinder (v.2.2.1) (40) with the parameters “-S diamond -M msa.” Gene search and annotation of MAGs analyzed in this study were performed using the Kyoto Encyclopedia of Genes and Genomes (KEGG) (41), followed by searching against Clusters of Orthologous Groups of proteins (COG) (42), the Protein families database (Pfam) (43), the Conserved Domain Database (CDD) (44), the Protein Clusters (PRK) (https://www.ncbi.nlm.nih.gov/proteinclusters/), the Institute for Genomic Research’s database of protein FAMilies (TIGRFAM) (45), the carbohydrate-active enzymes database (CAZy) (46), the bacterial peptidases database (MEROPS) (47), and the NCBI non-redundant (nr) database using the BLASTP program (*e*-value <1 × 10^–5^, identity >30%, query coverage >50%) (48). Extracellular peptidases were predicted using SignalP (v.6.0) (49) and PSORTb (v.3.0.3) (50). The metabolic pathways were reconstructed based on the above-predicted annotations, and reference pathways were depicted in KEGG and MetaCyc (51). A detailed workflow was shown in Fig. S2. Taxonomic classification of each protein-coding gene in the *nifH*-containing contig of *Ketobacter* W.bin6.184 was identified using CAT tool (v.6.0.1) (52) against NCBI reference protein database.

### Phylogenetic analysis and HGT gene identification

The phylogenetic trees of *nxrA*, *aclA*, *aclB*, *rbcL*, *nifH*, and cytochrome *c* oxidase genes were constructed using IQ-TREE (v.1.6.3) (53) with ModelFinder (54), and ultrafast bootstrapping was used to estimate the reliability of each branch with 1,000 times resampling. The reference sequences with BLASTP identity >30% to the target genes were retrieved from the NCBI nr database (37) and the UniProt database (55). The phylogenomic tree of anammox bacteria was constructed using IQ-TREE with ModelFinder, based on the 120 conserved single-copy ubiquitous bacterial genes (33). The trees were visualized using the iTOL online tool (v.6) (56). The HGT genes were predicted by the HGTector tool (v.2.0b3) (57) and the genes encoding type A cytochrome *c* oxidase in D200.bin4.133 were analyzed by constructing a phylogenetic tree using IQ-TREE.

## RESULTS

### Microbial community structures in the MT biosphere

**Fig 1:**
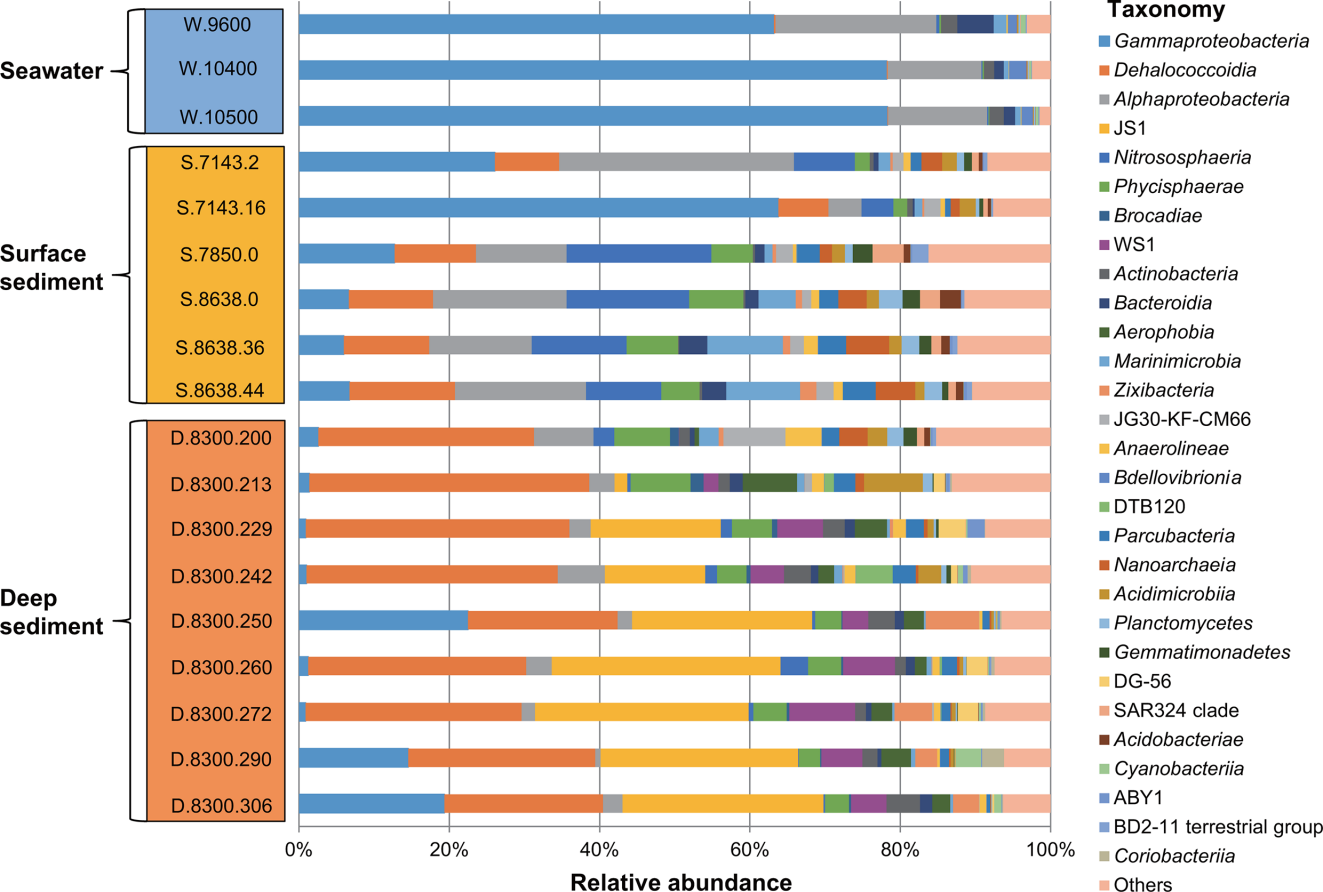
Relative abundances of dominant microbial groups based on 16S rRNA gene sequences retrieved from the clean reads of each metagenome. A relative abundance of less than 2% was grouped into others. Detailed values are provided in Table S2.

A detailed survey based on the 16S rRNA gene sequences retrieved from the clean reads of the metagenome revealed 348, 222, and 139 microbial classes in the deep sediments, surface sediments, and seawater of the MT, respectively (Fig. 1; Table S2). The three habitats all showed a dominance of bacteria over archaea (>84.5% bacteria in relative abundance). *Proteobacteria*, *Chloroflexota*, *Planctomycetota,* and *Thermoproteota* (represented by *Nitrososphaeria*) were common microbial groups in the MT sediments and seawater. However, distinct dominant microbial taxa were observed in the three habitats (Fig. 1; Table S2). For example, *Dehalococcoidia* (*Chloroflexota*) (>19%) and JS1 (*Atribacterota*) (>1.6%) were the predominant microbial groups in the deep sediments. The highest relative abundances of *Dehalococcoidia* occurred in each sample of D.8300.200 (28.6%), D.8300.213 (37.0%), D.8300.229 (34.5%), D.8300.242 (31.7%), and D.8300.272 (28.2%). The JS1 bacteria had the highest relative abundance in each sample of D.8300.250 (23.4%), D.8300.260 (29.9%), D.8300.290 (25.2%), and D.8300.306 (25.5%). Interestingly, the average relative abundance of *Brocadiae* (*Planctomycetota*) in the MT deep sediments (200–306 cmbsf) was significantly higher than that in surface sediments (0–46 cmbsf) (0.57% versus 0.03%; Mann-Whitney U test, *P* < 0.01).

*Alphaproteobacteria* was a major group in the surface sediment at S.7143.2 (31.1%), S.7143.16 (4.4%), S.7850.0 (12.0%), S.8638.0 (17.7%), S.8638.36 (13.6%), and S.8638.44 (17.4%). Furthermore, a higher relative abundance of *Nitrososphaeria* was detected in the surface sediment compared with the deep sediment or seawater. In the seawater, the major microbial groups included *Gammaproteobacteria* (60.3-73.7%) and *Alphaproteobacteria* (11.8%–20.4%).

### Diverse microbial nitrogen cycling processes

**Fig 2:**
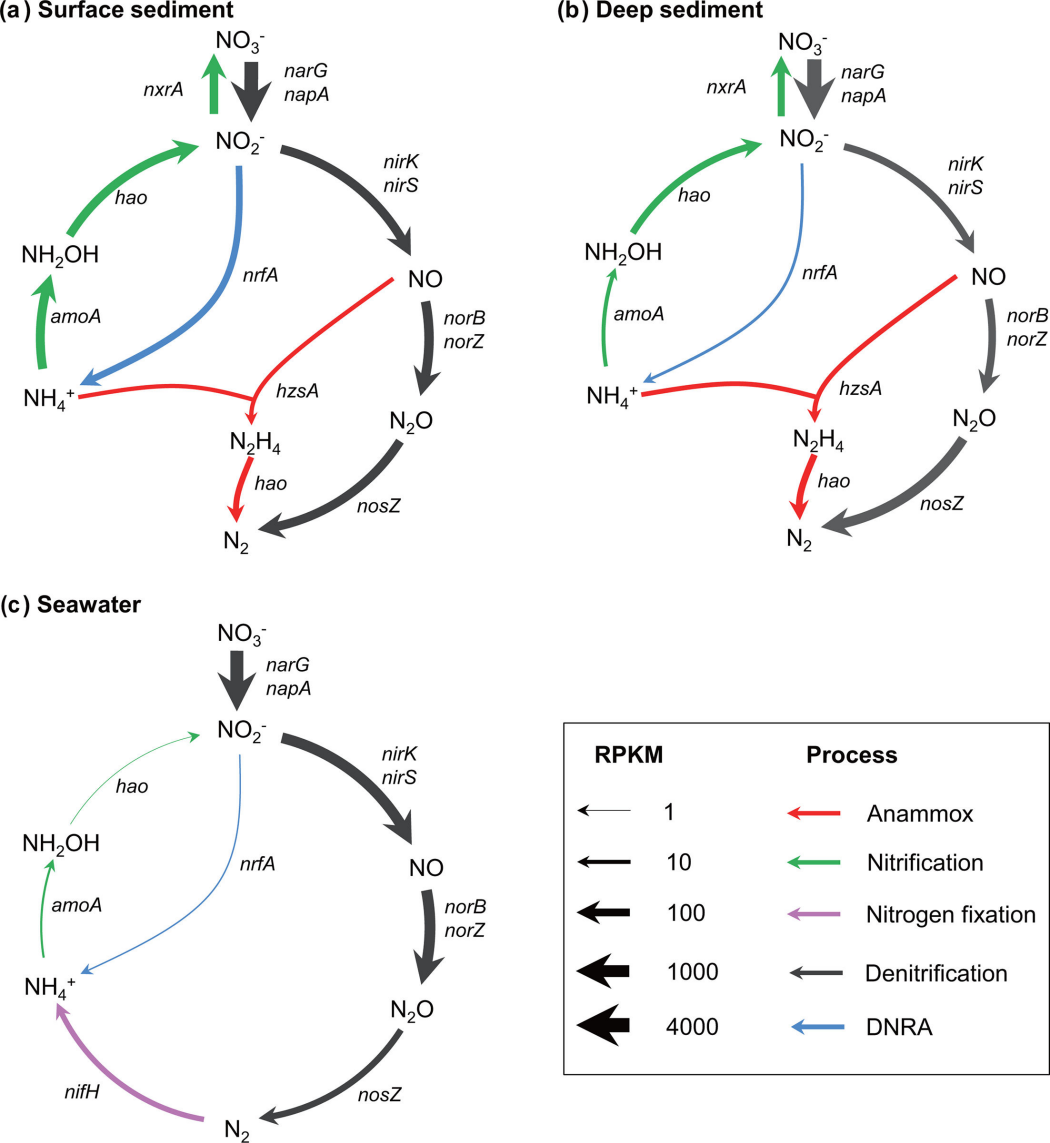
Microbial nitrogen cycling networks in the (**a**) surface sediment, (**b**) deep sediment, and (**c**) seawater of the MT. The thickness of each line represents the average abundance (expressed as RPKM) of the key genes in the pathway. Different colors represent different microbial nitrogen processes. *narG*, membrane-bound nitrate reductase alpha subunit; *napA*, periplasmic nitrate reductase; *nrfA*, periplasmic cytochrome *c* nitrite reductase, *nirS*, heme-containing nitrite reductase; *nirK*, Cu-containing nitrite reductase; *norB*, cytochrome *c*-dependent nitric oxide reductase subunit B; *norZ*, quinol-dependent nitric oxide reductase; *nosZ*, nitrous oxide reductase; *amoA*, ammonia monooxygenase subunit A; *hao*, hydroxylamine oxidoreductase/hydrazine dehydrogenase; *nxrA*, nitrite oxidoreductase alpha subunit; *hzsA*, hydrazine synthase subunit A; *nifH*, molybdenum-iron nitrogenase. Detailed values are provided in Table S3.

In general, various microorganisms contain key genes that participate in nitrogen cycling processes, including DNRA [membrane-bound nitrate reductase (NAR, *narG*), periplasmic nitrate reductase (NAP, *napA*), periplasmic cytochrome nitrite reductase (ccNIR, *nrfA*)], denitrification [NAR, NAP, heme-containing nitrite reductase (cd_1_-NIR, *nirS*), Cu-containing nitrite reductase (Cu-NIR, *nirK*), cytochrome *c*-dependent nitric oxide reductase (cNOR, *norB*), quinol-dependent nitric oxide reductase (qNOR, *norZ*), nitrous oxide reductase (NOS, *nosZ*)], nitrification [ammonia monooxygenase (AMO, *amoA*), hydroxylamine oxidoreductase (HAO, *hao*), nitrite oxidoreductase (NXR, *nxr*)], anammox [hydrazine dehydrogenase (HAO, *hao*), hydrazine synthase (HZS, *hzs*)], and nitrogen fixation [molybdenum-iron nitrogenase (MoFe, *nifH*)] (17, 22). The abundances of functional genes [expressed as reads per kilobase per million sequenced reads (RPKM)] in the denitrification pathway (*nirK*, *norBC*) were higher in the surface sediments than in the deep sediments. An abundance of genes in the DNRA or denitrification process (*napA*/*narG*, *nrfA*) was higher in the surface sediments than in the seawater (Fig. 2; Table S3; Mann-Whitney U test, *P* < 0.05). The MT seawater metagenomes also showed higher abundances of *nirK* and *norB* genes, as compared with those in the deep sediments (Mann-Whitney U test, *P* < 0.05). However, the abundance of *nosZ* gene in the deep sediments was eightfold higher than in seawater (Mann-Whitney U test, *P* < 0.05). Notably, unique microbial nitrogen cycling genes were detected in the three habitats, including nitrite oxidation and anammox in the sediments, and nitrogen fixation in the seawater.

#### Newly-identified NOB and anammox bacteria in the MT sediment

**Fig 3:**
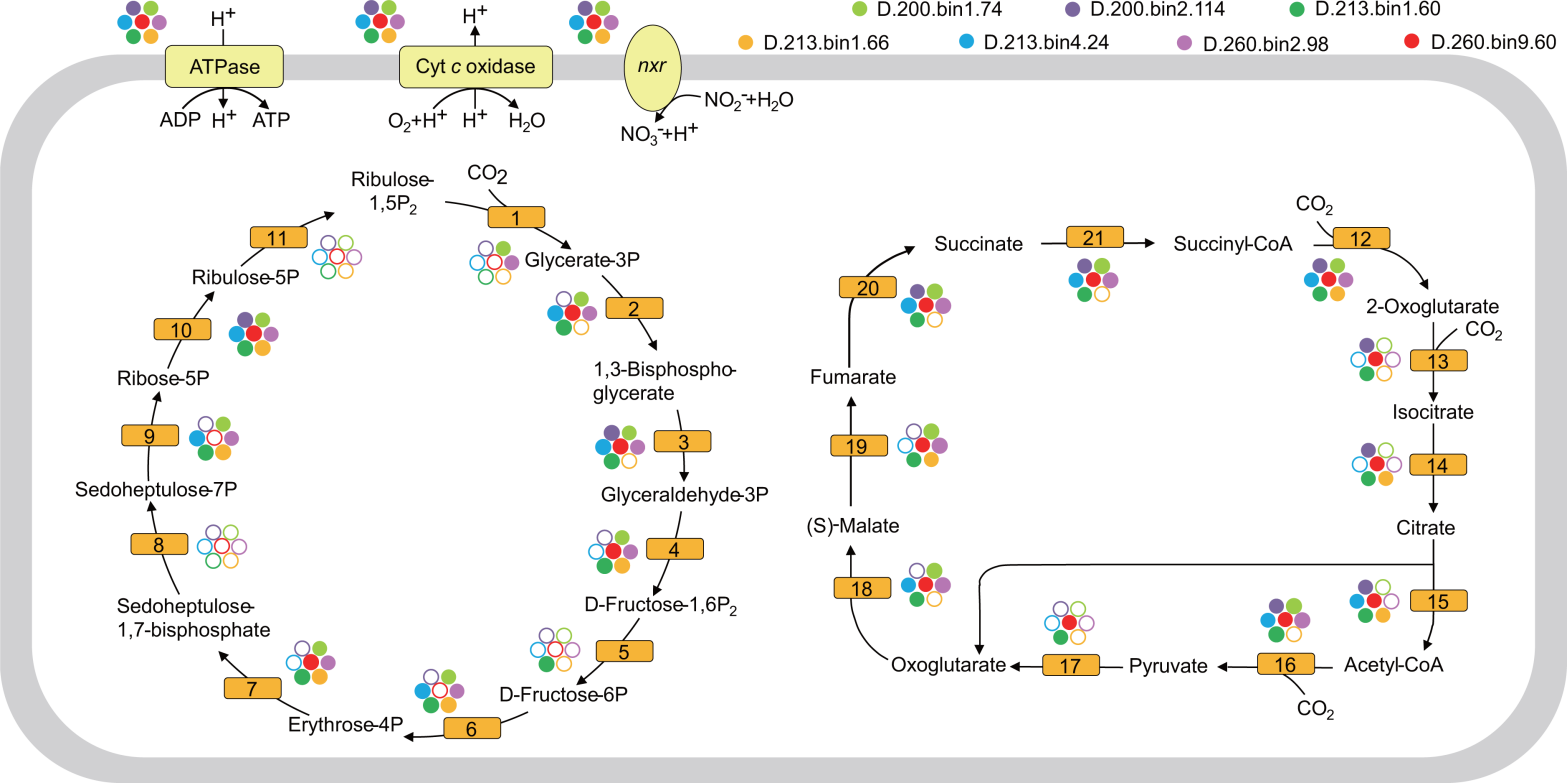
Reconstructed metabolic pathways of seven NOB MAGs in the MT deep sediment. Each filled or hollow circle indicates that the gene is present or absent in the MAG, respectively. A full list of genes labeled with different letters is provided in Table S5.

Nitrite oxidation was a significant microbial nitrogen process in the MT sediment compared with seawater because the metagenomes in the surface and deep sediments contained the hallmark gene nitrite oxidoreductase (*nxr*) (average RPKM value of 149 and 60, respectively; Fig. 2; Fig. S3). Of note, seven nitrite oxidoreductase-containing genomes were recovered from the metagenomes of the MT deep sediment. These MAGs were assigned to taxa including *Desulfobacterota*, *Latescibacterota*, *Omnitrophota*, *Planctomycetota*, and unclassified bacteria JABMQX01 according to the taxonomy of Genome Taxonomy Database (GTDB) (Table S4), which are distinct from the common NOB taxa (e.g., *Chloroflexota*, *Proteobacteria*, *Nitrospinota*, and *Nitrospirota*) as described in previous studies (58). Furthermore, these NOBs could potentially fix inorganic carbon because the complete gene sets in the rTCA or CBB cycles were identified in the NOB genomes, including the key genes encoding ATP citrate lyase (*acl*) or ribulose-bisphosphate carboxylase (*rbc*), respectively (Fig. 3; Fig. S4 through S6; Table S5).

**Fig 4:**
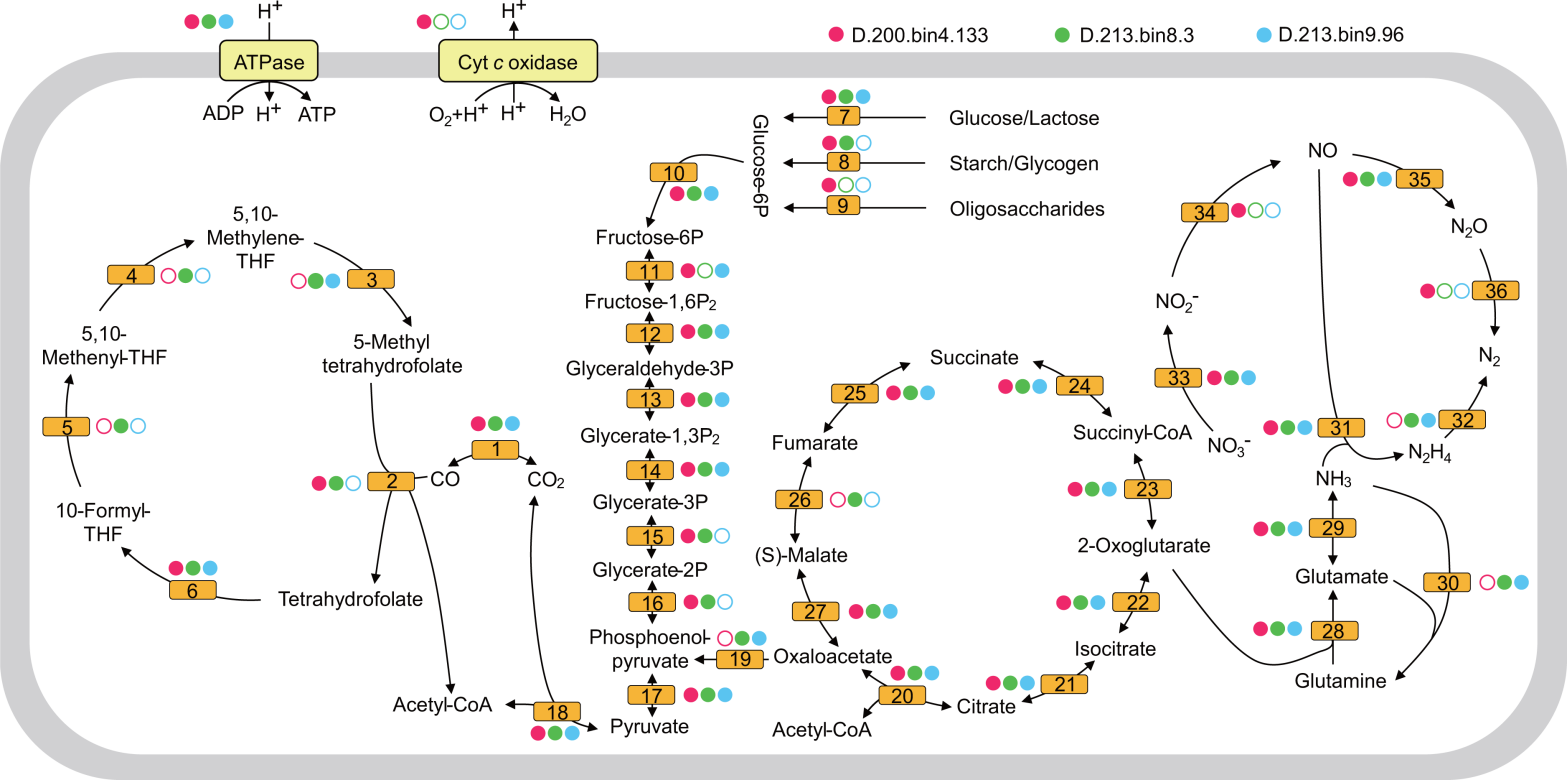
Reconstructed metabolic pathways of three anammox bacteria MAGs in the MT deep sediment. Each filled or hollow circle indicates that the gene is present or absent in the MAG, respectively. A full list of genes labeled with different numbers is provided in Table S6.

Furthermore, anammox, determined by the presence of the hydrazine synthase gene (*hzs*), only occurred in the MT sediment (Fig. 2). Three anammox MAGs were then retrieved from the metagenomes of the MT deep sediments, among which D.213.bin9.96 and D.200.bin4.133 belonged to *Scalinduaceae*, and D.213.bin8.3 belonged to the recently named *Bathyanammoxibiaceae* (Fig. S6). To reveal the characteristics of anammox bacteria in the MT deep sediment, we performed the comparative genomic analysis of 76 anammox bacteria genomes (>50% completeness and <5% contamination), including 3 MAGs retrieved from this study (59, 60), 69 MAGs from the NCBI Assembly database (37), and 4 MAGs from the genomic catalog of Earth’s microbiomes (38). Interestingly, some genomes from *Bathyanammoxibiaceae*, *Scalinduaceae,* and *Brocadiaceae* contained genes encoding cytochrome *c* oxidases (Fig. S7). A phylogenetic analysis of the cytochrome *c* oxidase large subunit genes showed that D.200.bin4.133 belonged to type A cytochrome *c* oxidase (*aa3*) and that genes in the other anammox bacteria were type C cytochrome *c* oxidase (*cbb3*) (Fig. S8). In addition, complete gene sets of the glycolysis pathway were detected in the three anammox MAGs from the MT deep sediments, including those encoding enzymes that oxidize multiple carbohydrates such as glucose, lactose, starch/glycogen, and oligosaccharides (Fig. 4; Table S6).

#### N_2_ fixers in the MT seawater

**Fig 5:**
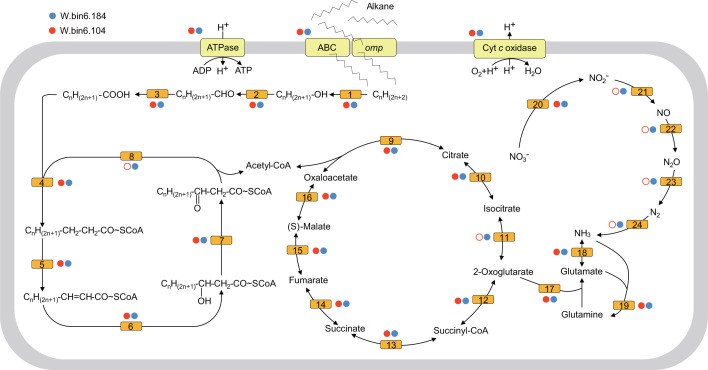
Reconstructed metabolic pathways of two *Ketobacter* MAGs in the MT seawater. Each filled or hollow circle indicates that the gene is present or absent in the MAG, respectively. A full list of genes labeled with different numbers is provided in Table S8.

The key gene *nifH*, which encodes nitrogenase, was detected exclusively in the MT seawater, suggesting the existence of microorganisms that convert inert N_2_ to bioavailable ammonia. Furthermore, the metagenomic binning of the three seawater samples recovered a *nifH*-containing MAG W.bin6.184. The MAG was assigned to the genus *Ketobacter* of the family *Alcanivoracaceae*, and its average relative abundance was 4.0% in the MT seawater (Fig. 2c; Fig. S9; Table S4). Meanwhile, all of the protein-coding genes in the *nifH*-containing contig of W.bin6.184 were assigned to *Ketobacter* (Table S7). To assess the metabolic features of W.bin6.184, we performed a comparative genomic analysis of 29 *Ketobacter* genomes (>50% completeness and <5% contamination) retrieved from this study (W.bin6.184, W.bin6.104), the NCBI Assembly database (20 MAGs) (37), and the genomic catalog of Earth’s microbiomes (7 MAGs) (38). Notably, a key gene alkane 1-monooxygenase (*alkB*), which is involved in the first step of medium-chain alkane oxidation, was detected in the two *Ketobacter* MAGs in this study (Fig. 5). In addition, complete gene sets involved in β-oxidation, the tricarboxylic acid cycle, and cytochrome *c* oxidases were present in the MAGs of W.bin6.184 (completeness 98.89%, contamination 2.39%) and W.bin6.104 (completeness 80.90%, contamination 0.65%) (Fig. 5; Table S8).

## DISCUSSION

### Distinct microbial communities exist the different MT environments

Due to the funneling effect of the hadal trench, the abundant organics accumulate in the MT trench that harbors abundant microorganisms (6). *Proteobacteria* (represented by *Gammaproteobacteria* and *Alphaproteobacteria*) and *Thaumarchaeota* (represented by *Nitrososphaeria*) were the most abundant bacterial and archaeal groups in the MT seawater and surface sediments, respectively (Fig. 1) (6, 25, 26). *Dehalococcoidia* and JS1 (*Atribacterota*) showed the highest relative abundances in the deep MT sediments, as compared with those in the seawater and surface sediments (Fig. 1; Mann-Whitney U test, *P* < 0.05). These results were in accordance with the deep sediments of the eastern equatorial Pacific and the North Pacific gyre, indicating that these bacterial groups might degrade halogenated organic compounds and aromatics present in the deep MT sediments (13, 61, 62).

### Five new NOB lineages fix CO_2_ in the MT sediment

Compared with sediment from the adjacent trench bottom and abyssal plain, the sediment layer of the MT slope is generally thinner with a lower organic carbon content (4, 6). Such an environment might favor the growth of chemolithoautotrophic microorganisms, such as AOA and NOB. In general, AOA is a dominant archaeal group in many marine sedimentary environments (63). However, NOB lineages are often overlooked because of the low NO_2_^−^ concentration and its rapid turnover rate in the sediments (61, 64, 65). Previous studies reported that NOBs have a very high affinity for NO_2_^−^ and that they are the predominant fixers of dissolved inorganic carbon in the dark ocean (64, 66). In the sediment of the MT slope, the average gene abundance of *nxrA* was comparable with that of *norBZ* (Fig. 2), suggesting that nitrite oxidation might be an important process that shares similar activity with NO reduction in the predominant denitrification pathway. The retention of nitrogen *via* nitrite oxidation (NO_2_^−^→NO_3_^−^) reduces the loss of nitrogen *via* denitrification (NO_2_^−^→NO) (67). However, the source of NO_2_^−^ in the MT sediments is unknown because the concentration of NO_2_^−^ was below the detection limit (<0.1 µM) (10). A recent study showed that the microbial nitrate reduction process was stimulated in the MT sediments (68), suggesting that NO_2_^−^ is supplied to the sediment through the microbial nitrate reduction process, or from the surrounding environment (58, 69).

Seven NOB genomes were recovered from the metagenomes of the MT deep sediments (Fig. 3; Fig. S3). These MAGs were identified as new NOB lineages that are not classified into the common NOB taxa *Nitrospinota*, *Nitrospirota*, *Chloroflexota*, or *Proteobacteria*. Of note, each of the seven new NOBs could fix inorganic carbon through the rTCA or CBB cycle (Fig. 3) (70). However, the O_2_ content in the MT sediment would be much lower than that in the surrounding seawater, indicating that these hadal NOBs had a microaerophilic lifestyle and entered the hadal sediment through physical setting or biological processes as similar to the NOB from *Nitrospira* (58, 71). As a result, the identification of these new NOBs not only extends their phylogenetic diversity but also reveals that nitrite oxidation might be an important yet overlooked microbial carbon fixation process in hadal sediment (71).

### Anammox bacteria perform aerobic respiration in the MT sediment

The identification of the *hzs* gene and the recovery of three anammox MAGs in the MT sediment confirm previous results reporting that anammox is an important nitrogen removal process in hadal sedimentary environments (6, 24, 61). Of note, the anammox bacterium (D.200.bin4.133) could perform aerobic respiration because it contained a gene encoding the type A cytochrome *c* oxidase (Fig. S7). There are three types of cytochrome *c* oxidases (type A, B, and C), of which type A cytochrome *c* oxidase mainly functions in high oxygen concentration environments compared with the other two types (72). Type A cytochrome *c* oxidase is also the terminal oxidase with the highest efficiency for energy generation (73). In some bacterial strains containing type A and type C cytochrome *c* oxidases (e.g., *Pseudomonas aeruginosa* PAO1 and *Shewanella oneidensis* MR-1), type A is highly expressed whereas type C remains unchanged under nutrient-limiting conditions (74, 75). This implies that compared with type C cytochrome *c* oxidase, type A cytochrome *c* oxidase is more adapted to oligotrophic environments such as the MT deep sediment. In addition, a phylogenetic tree containing a collection of 229 type A cytochrome *c* oxidase reference genes revealed that D.200.bin4.133 might have acquired the type A cytochrome *c* oxidase gene from *Desulfobacterota* by horizontal gene transfer (Fig. S10).

Furthermore, the metabolic pathway reconstruction of the anammox bacterium D.200.bin4.133 showed that this strain can obtain energy by utilizing multiple carbohydrates (glucose, lactose, starch/glycogen, or oligosaccharides) (Fig. 4), which would provide more ATPs than anaerobic metabolism when O_2_ is the terminal electron acceptor (76, 77). Anammox bacteria have demonstrated their ability to live in oxygenated environments. For example, “*Candidatus* Brocadia caroliniensis” and “*Candidatus* Kuenenia stuttgartiensis” were observed to tolerate oxygen concentrations of up to 120 µM and 200 µM, respectively (78). The defense mechanisms against oxygen in these anammox bacteria may be attributed to the expression of enzymes such as bilirubin oxidase, cytochrome *c* oxidase, and bifunctional catalase-peroxidase (79). Similarly, the gene encoding type A cytochrome *c* oxidase in the genome of D.200.bin4.133 might act as an oxygen scavenger to maintain the anammox process in an anaerobic environment, which confers a selective advantage to D.200.bin4.133 that faces O_2_ concentration fluctuations in MT (4, 80, 81). The O_2_ content in the MT seawater below 6,000 mbsl was constantly within the range between 156 µM and 188 µM (10). The source of O_2_ in the MT sediment may be derived from the overlying seawater that penetrates through the seawater-sediment interface (6, 82). Moreover, the abundant AOA species in the MT sediment might also produce a small amount of O_2_ as reported recently (83, 84), and the O_2_ could be rapidly utilized by other aerobic microorganisms such as anammox bacterium D.200.bin4.133. However, more evidence is needed to test the activity of aerobic respiration of MT anammox bacteria, such as metatranscriptomics, metaproteomics, and rate measurements.

### Alkane-degrading *Ketobacter* fix N_2_ in the MT seawater

N_2_ production by microbial denitrification and anammox processes forms the largest nitrogen sink in the ocean (24, 85). Recent studies revealed that most of N_2_ is produced *via* the anammox process in the hadal sediments of the Atacama Trench (~67%) and Kermadec Trench (>90%) (6, 24). By contrast, in the MT seawater, the denitrification process is responsible for most N_2_ production because nitrate (~36 µM) and denitrifying microorganisms are abundant (Fig. 2) (10, 25). The accumulated N_2_ is then used by an abundant nitrogenase-containing *Ketobacter* strain W.bin6.184 (Fig. 5). However, N_2_ fixation is an extremely high energy-consuming process (16 ATPs per mole of N_2_ fixed) (86, 87). Various organic matter could serve as energy sources for nitrogen fixation, such as cellulose, chitin, glucan, pectin, polyphenols, starch, and alkane (88, 89). To fulfill this large energy requirement, *Ketobacter* W.bin6.184 might have the potential to perform the aerobic degradation of medium-chain alkanes to produce acetyl-CoA (>100 ATPs per mole of medium-chain alkane oxidized) (90) because of the presence of complete gene sets for alkane oxidation (*alk* and β-oxidation) (Fig. 5). This is consistent with the features of most of the genera in *Alcanivoracaceae*, a well-known aerobic hydrocarbon-degrading bacterial family (91, 92). Of note, the *alkB* gene was detected in most of the *Ketobacter* genomes (Table S8), and the utilization of n-alkane by a pure *Ketobacter* strain was demonstrated by incubation experiments (91), suggesting that *Ketobacter* W.bin6.184 could obtain energy from alkane degradation. The concentration of n-alkanes was 23.5  µg/gdw in the MT seawater as measured previously (26, 93), and the alkane-degrading bacteria including the members of *Alcanivoracaceae* were abundant in the MT seawater (Fig. 1). Possible sources of the alkanes in the MT might include a mixture of biological processes (through rotting organisms) and geological processes (through water-rock reactions) (94, 95). The slow degradation of alkanes may spread long distances with the effects of hadal seawater currents (94).

### Conclusion

**Fig 6:**
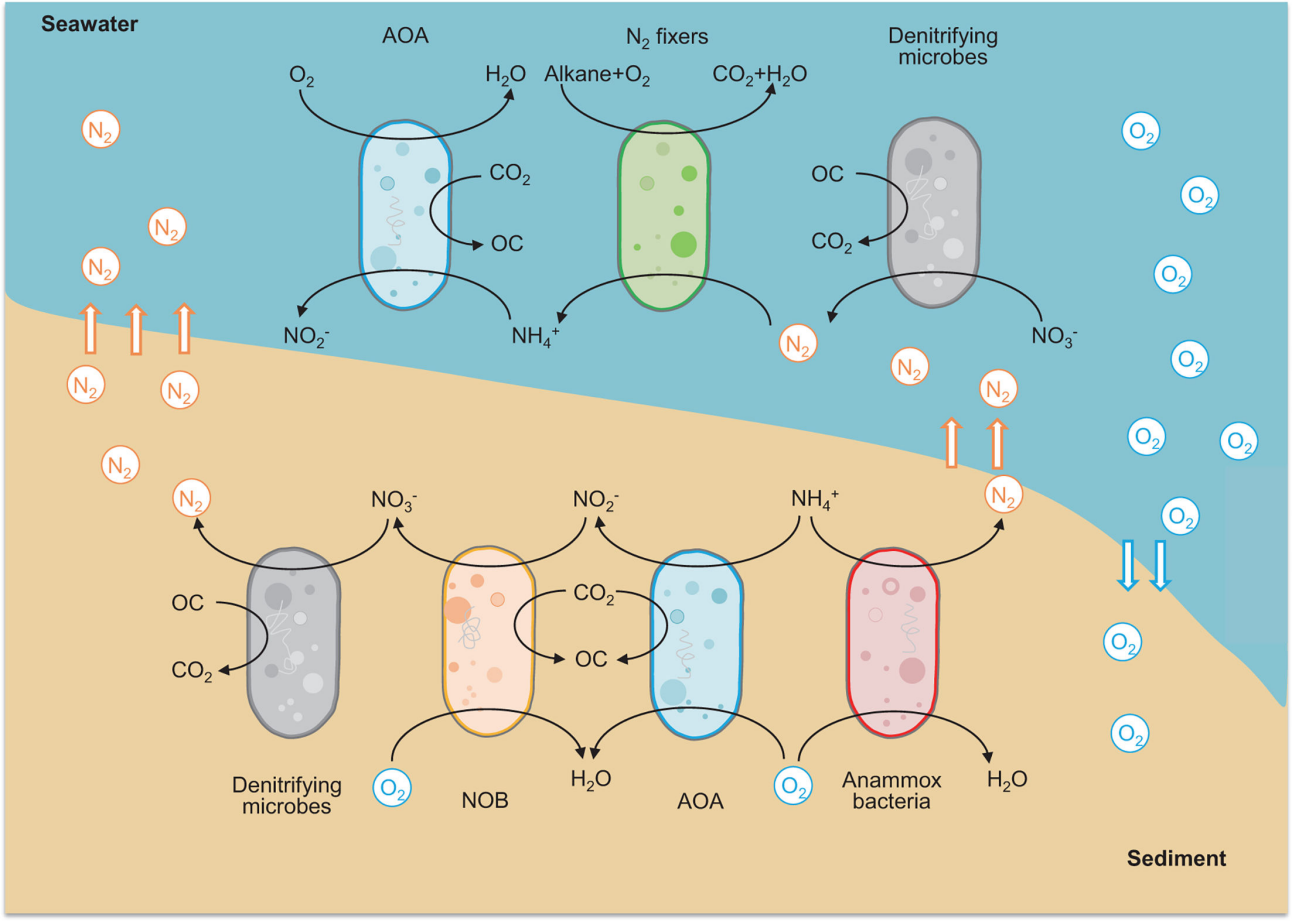
Schematic representation of the microbial nitrogen cycling processes in the MT sediment and seawater. OC, organic carbon. Detailed information on the MAGs belonging to AOA, NOB, anammox bacteria, N_2_ fixers, and denitrifying microbes is provided in Table S4.

This study provides new insights into the unique features of the microbial nitrogen cycling processes in the deepest part of the ocean. The distinct dominant microbial taxa were observed in the different MT habitats. The identification of five new NOB lineages in the MT sediment has uncovered an overlooked process for inorganic carbon fixation. Meanwhile, anammox bacteria might perform aerobic respiration in response to nutrient limitations or O_2_ fluctuations in the sediment. In the MT seawater, an abundant *Ketobacter* strain might obtain energy during alkane degradation, and then fix N_2_ released from sedimentary denitrifiers and anammox bacteria (Fig. 6). The integration of multi-omics strategy that combines metagenomics with metatranscriptomics, metaproteomics, or metabolomics will provide more comprehensive understanding of hadal microbial communities in future studies. Meanwhile, laboratory incubation experiments and *in situ* activity tests are also needed to verify the contribution of hadal microbial communities to the global nitrogen biogeochemical cycles.

## Supplementary Material

Reviewer comments

## Data Availability

The genome sequences from the current study have been deposited in eLMSG (an eLibrary of Microbial Systematics and Genomics, https://www.biosino.org/elmsg/) under accession numbers (MAGs) LMSG_G000011485.1-LMSG_G000011652.1. Raw reads of the metagenomes for MT deep sediments have been deposited in NODE (The National Omics Data Encyclopedia, https://www.biosino.org/node/) under the accession number OES290491-OES290499.

## SUPPLEMENTAL MATERIAL

Supplemental Figures msystems.00243-24-s0001.pdf Figures S1 to S10.

Supplemental Tables msystems.00243-24-s0002.xlsx Tables S1 to S8.

OPEN PEER REVIEW reviewer-comments.pdf An accounting of the reviewer comments and feedback.

ASM does not own the copyrights to Supplemental Material that may be linked to, or accessed through, an article. The authors have granted ASM a non-exclusive, world-wide license to publish the Supplemental Material files. Please contact the corresponding author directly for reuse.

## References

[1] Jamieson AJ, Fujii T, Mayor DJ, Solan M, Priede IG. 2010. Hadal trenches: the ecology of the deepest places on Earth. Trends Ecol Evol 25:190–197. doi:10.1016/j.tree.2009.09.009 19846236

[2] Taira K, Yanagimoto D, Kitagawa S. 2005. Deep CTD casts in the challenger deep. J Oceanogr 61:447–454. doi:10.1007/s10872-005-0053-z

[3] Zhang X, Xu W, Liu Y, Cai M, Luo Z, Li M. 2018. Metagenomics reveals microbial diversity and metabolic potentials of seawater and surface sediment from a hadal biosphere at the Yap Trench. Front Microbiol 9:2402. doi:10.3389/fmicb.2018.02402 30369913 PMC6194347

[4] Glud RN, Wenzhöfer F, Middelboe M, Oguri K, Turnewitsch R, Canfield DE, Kitazato H. 2013. High rates of microbial carbon turnover in sediments in the deepest oceanic trench on Earth. Nature Geosci 6:284–288. doi:10.1038/ngeo1773

[5] Luo M, Gieskes J, Chen LY, Shi XF, Chen DF. 2017. Provenances, distribution, and accumulation of organic matter in the southern Mariana Trench rim and slope: implication for carbon cycle and burial in hadal trenches. Marine Geology 386:98–106. doi:10.1016/j.margeo.2017.02.012

[6] Zhou YL, Mara P, Cui GJ, Edgcomb VP, Wang Y. 2022. Microbiomes in the Challenger Deep slope and bottom-axis sediments. Nat Commun 13:1515. doi:10.1038/s41467-022-29144-4 35314706 PMC8938466

[7] Jamieson AJ, Stewart HA. 2021. Hadal zones of the Northwest Pacific Ocean. Prog Oceanogr 190:102477. doi:10.1016/j.pocean.2020.102477

[8] Jamieson AJ, Stewart HA, Rowden AA, Clark MR. 2020. Geomorphology and benthic habitats of the Kermadec Trench, Southwest Pacific Ocean, p 949–966. In Harris PT, Baker E (ed), Seafloor geomorphology as benthic habitat, 2nd ed. Elsevier Science.

[9] Cui GJ, Li J, Gao ZM, Wang Y. 2019. Spatial variations of microbial communities in abyssal and hadal sediments across the Challenger Deep. PeerJ 7:e6961. doi:10.7717/peerj.6961 31149407 PMC6526897

[10] Nunoura T, Takaki Y, Hirai M, Shimamura S, Makabe A, Koide O, Kikuchi T, Miyazaki J, Koba K, Yoshida N, Sunamura M, Takai K. 2015. Hadal biosphere: insight into the microbial ecosystem in the deepest ocean on Earth. Proc Natl Acad Sci U S A 112:E1230–6. doi:10.1073/pnas.1421816112 25713387 PMC4371994

[11] Peoples LM, Grammatopoulou E, Pombrol M, Xu X, Osuntokun O, Blanton J, Allen EE, Nunnally CC, Drazen JC, Mayor DJ, Bartlett DH. 2019. Microbial community diversity within sediments from two geographically separated hadal trenches. Front Microbiol 10:347. doi:10.3389/fmicb.2019.00347 30930856 PMC6428765

[12] Zhong HH, Lehtovirta-Morley L, Liu JW, Zheng YF, Lin HY, Song DL, Todd JD, Tian JW, Zhang XH. 2020. Novel insights into the Thaumarchaeota in the deepest oceans: their metabolism and potential adaptation mechanisms. Microbiome 8:78. doi:10.1186/s40168-020-00849-2 32482169 PMC7265257

[13] Liu RL, Wei X, Song WZ, Wang L, Cao JW, Wu JW, Thomas T, Jin T, Wang ZX, Wei WX, Wei YL, Zhai HF, Yao C, Shen ZY, Du JT, Fang JS. 2022. Novel Chloroflexi genomes from the deepest ocean reveal metabolic strategies for the adaptation to deep-sea habitats. Microbiome 10:75. doi:10.1186/s40168-022-01263-6 35538590 PMC9088039

[14] Zhong HH, Sun H, Liu RH, Zhan YC, Huang XY, Ju F, Zhang XH. 2021. Comparative genomic analysis of Labrenzia aggregata (Alphaproteobacteria) strains isolated from the Mariana Trench: insights into the metabolic potentials and biogeochemical functions. Front Microbiol 12:770370. doi:10.3389/fmicb.2021.770370 34970235 PMC8712697

[15] Abdel-Mageed WM, Juhasz B, Lehri B, Alqahtani AS, Nouioui I, Pech-Puch D, Tabudravu JN, Goodfellow M, Rodríguez J, Jaspars M, Karlyshev AV. 2020. Whole genome sequence of Dermacoccus abyssi MT1.1 isolated from the Challenger Deep of the Mariana Trench reveals phenazine biosynthesis locus and environmental adaptation factors. Mar Drugs 18:131. doi:10.3390/md18030131 32106586 PMC7143476

[16] Zheng Y, Wang J, Zhou S, Zhang Y, Liu J, Xue C-X, Williams BT, Zhao X, Zhao L, Zhu X-Y, Sun C, Zhang H-H, Xiao T, Yang G-P, Todd JD, Zhang X-H. 2020. Bacteria are important dimethylsulfoniopropionate producers in marine aphotic and high-pressure environments. Nat Commun 11:4658. doi:10.1038/s41467-020-18434-4 32938931 PMC7494906

[17] Kuypers MMM, Marchant HK, Kartal B. 2018. The microbial nitrogen-cycling network. Nat Rev Microbiol 16:263–276. doi:10.1038/nrmicro.2018.9 29398704

[18] Takai K. 2019. The nitrogen cycle: a large, fast, and mystifying cycle. Microbes Environ 34:223–225. doi:10.1264/jsme2.ME3403rh 31554776 PMC6759337

[19] Vuillemin A, Wankel SD, Coskun ÖK, Magritsch T, Vargas S, Estes ER, Spivack AJ, Smith DC, Pockalny R, Murray RW, D’Hondt S, Orsi WD. 2019. Archaea dominate oxic subseafloor communities over multimillion-year time scales. Sci Adv 5:eaaw4108. doi:10.1126/sciadv.aaw4108 31223656 PMC6584578

[20] Peoples LM, Donaldson S, Osuntokun O, Xia Q, Nelson A, Blanton J, Allen EE, Church MJ, Bartlett DH. 2018. Vertically distinct microbial communities in the Mariana and Kermadec trenches. PLoS One 13:e0195102. doi:10.1371/journal.pone.0195102 29621268 PMC5886532

[21] Nunoura T, Nishizawa M, Hirai M, Shimamura S, Harnvoravongchai P, Koide O, Morono Y, Fukui T, Inagaki F, Miyazaki J, Takaki Y, Takai K. 2018. Microbial diversity in sediments from the bottom of the Challenger Deep, the Mariana Trench. Microbes Environ 33:186–194. doi:10.1264/jsme2.ME17194 29806625 PMC6031389

[22] Chen P, Zhou H, Huang YY, Xie Z, Zhang MJ, Wei YL, Li J, Ma YW, Luo M, Ding WM, Cao JW, Jiang T, Nan P, Fang JS, Li X. 2021. Revealing the full biosphere structure and versatile metabolic functions in the deepest ocean sediment of the Challenger Deep. Genome Biol 22:207. doi:10.1186/s13059-021-02408-w 34256809 PMC8276468

[23] Jing HM, Xiao X, Zhang Y, Li ZY, Jian HH, Luo YF, Han Z. 2022. Composition and ecological roles of the core microbiome along the abyssal-hadal transition Zone sediments of the Mariana Trench. Microbiol Spectr 10:e0198821. doi:10.1128/spectrum.01988-21 35768947 PMC9241748

[24] Thamdrup B, Schauberger C, Larsen M, Trouche B, Maignien L, Arnaud-Haond S, Wenzhöfer F, Glud RN. 2021. Anammox bacteria drive fixed nitrogen loss in hadal trench sediments. Proc Natl Acad Sci U S A 118:e2104529118. doi:10.1073/pnas.2104529118 34764222 PMC8609620

[25] Xue CX, Liu JW, Lea-Smith DJ, Rowley G, Lin H, Zheng YF, Zhu XY, Liang JC, Ahmad W, Todd JD, Zhang XH. 2020. Insights into the vertical stratification of microbial ecological roles across the deepest sseawater column on Earth. Microorganisms 8:1309. doi:10.3390/microorganisms8091309 32867361 PMC7565560

[26] Liu JW, Zheng YF, Lin HY, Wang X, Li M, Liu Y, Yu M, Zhao MX, Pedentchouk N, Lea-Smith DJ, Todd JD, Magill CR, Zhang WJ, Zhou S, Song DL, Zhong HH, Xin Y, Yu M, Tian JW, Zhang XH. 2019. Proliferation of hydrocarbon-degrading microbes at the bottom of the Mariana Trench. Microbiome 7:47. doi:10.1186/s40168-019-0652-3 30975208 PMC6460516

[27] Bolger AM, Lohse M, Usadel B. 2014. Trimmomatic: a flexible trimmer for illumina sequence data. Bioinformatics 30:2114–2120. doi:10.1093/bioinformatics/btu170 24695404 PMC4103590

[28] Prjibelski A, Antipov D, Meleshko D, Lapidus A, Korobeynikov A. 2020. Using SPAdes de novo assembler. CP in Bioinformatics 70:e102. doi:10.1002/cpbi.102 32559359

[29] Zhang X, Liu Z, Xu W, Pan J, Huang Y, Cai M, Luo Z, Li M. 2022. Genomic insights into versatile lifestyle of three new bacterial candidate phyla. Sci. China Life Sci 65:1547–1562. doi:10.1007/s11427-021-2037-x 35060074

[30] Kang DWD, Li F, Kirton E, Thomas A, Egan R, An H, Wang Z. 2019. MetaBAT 2: an adaptive binning algorithm for robust and efficient genome reconstruction from metagenome assemblies. PeerJ 7:e7359. doi:10.7717/peerj.7359 31388474 PMC6662567

[31] Sieber CMK, Probst AJ, Sharrar A, Thomas BC, Hess M, Tringe SG, Banfield JF. 2018. Recovery of genomes from metagenomes via a dereplication, aggregation and scoring strategy. Nat Microbiol 3:836–843. doi:10.1038/s41564-018-0171-1 29807988 PMC6786971

[32] Parks DH, Imelfort M, Skennerton CT, Hugenholtz P, Tyson GW. 2015. CheckM: assessing the quality of microbial genomes recovered from isolates, single cells, and metagenomes. Genome Res 25:1043–1055. doi:10.1101/gr.186072.114 25977477 PMC4484387

[33] Chaumeil PA, Mussig AJ, Hugenholtz P, Parks DH. 2019. GTDB-Tk: a toolkit to classify genomes with the Genome Taxonomy Database. Bioinformatics 36:1925–1927. doi:10.1093/bioinformatics/btz848 31730192 PMC7703759

[34] Bengtsson-Palme J, Hartmann M, Eriksson KM, Pal C, Thorell K, Larsson DGJ, Nilsson RH. 2015. METAXA2: improved identification and taxonomic classification of small and large subunit rRNA in metagenomic data. Mol Ecol Resour 15:1403–1414. doi:10.1111/1755-0998.12399 25732605

[35] Zhang M, Zhang X, Tran NT, Sun Z, Zhang X, Ye H, Zhang Y, Ma H, Aweya JJ, Li S. 2021. Molting alters the microbiome, immune response, and digestive enzyme activity in mud crab (Scylla paramamosain). mSystems 6:e0091721. doi:10.1128/mSystems.00917-21 34636669 PMC8510556

[36] Hiraoka S, Hirai M, Matsui Y, Makabe A, Minegishi H, Tsuda M, Rastelli E, Danovaro R, Corinaldesi C, Kitahashi T, Tasumi E, Nishizawa M, Takai K, Nomaki H, Nunoura T, Juliarni. 2020. Microbial community and geochemical analyses of trans-trench sediments for understanding the roles of hadal environments. ISME J 14:740–756. doi:10.1038/s41396-019-0564-z 31827245 PMC7031335

[37] Coordinators NR. 2016. Database resources of the National Center for Biotechnology Information. Nucleic Acids Res 44:D7–19. doi:10.1093/nar/gkv1290 26615191 PMC4702911

[38] Nayfach S, Roux S, Seshadri R, Udwary D, Varghese N, Schulz F, Wu D, Paez-Espino D, Chen I-M, Huntemann M, et al.. 2021. A genomic catalog of Earth's microbiomes. Nat Biotechnol 39:499–509. doi:10.1038/s41587-020-0718-6 33169036 PMC8041624

[39] Hyatt D, Chen GL, Locascio PF, Land ML, Larimer FW, Hauser LJ. 2010. Prodigal: prokaryotic gene recognition and translation initiation site identification. BMC Bioinformatics 11:119. doi:10.1186/1471-2105-11-119 20211023 PMC2848648

[40] Emms DM, Kelly S. 2019. OrthoFinder: phylogenetic orthology inference for comparative genomics. Genome Biol 20:238. doi:10.1186/s13059-019-1832-y 31727128 PMC6857279

[41] Kanehisa M, Sato Y, Kawashima M, Furumichi M, Tanabe M. 2016. KEGG as a reference resource for gene and protein annotation. Nucleic Acids Res 44:D457–62. doi:10.1093/nar/gkv1070 26476454 PMC4702792

[42] Tatusov RL, Fedorova ND, Jackson JD, Jacobs AR, Kiryutin B, Koonin EV, Krylov DM, Mazumder R, Mekhedov SL, Nikolskaya AN, Rao BS, Smirnov S, Sverdlov AV, Vasudevan S, Wolf YI, Yin JJ, Natale DA. 2003. The COG database: an updated version includes eukaryotes. BMC Bioinformatics 4:41. doi:10.1186/1471-2105-4-41 12969510 PMC222959

[43] Mistry J, Chuguransky S, Williams L, Qureshi M, Salazar GA, Sonnhammer ELL, Tosatto SCE, Paladin L, Raj S, Richardson LJ, Finn RD, Bateman A. 2021. Pfam: the protein families database in 2021. Nucleic Acids Res 49:D412–D419. doi:10.1093/nar/gkaa913 33125078 PMC7779014

[44] Lu SN, Wang JY, Chitsaz F, Derbyshire MK, Geer RC, Gonzales NR, Gwadz M, Hurwitz DI, Marchler GH, Song JS, Thanki N, Yamashita RA, Yang MZ, Zhang DC, Zheng CJ, Lanczycki CJ, Marchler-Bauer A. 2020. CDD/SPARCLE: the conserved domain database in 2020. Nucleic Acids Res 48:D265–D268. doi:10.1093/nar/gkz991 31777944 PMC6943070

[45] Haft DH, Selengut JD, Richter RA, Harkins D, Basu MK, Beck E. 2013. TIGRFAMs and genome properties in 2013. Nucleic Acids Res 41:D387–D395. doi:10.1093/nar/gks1234 23197656 PMC3531188

[46] Lombard V, Golaconda Ramulu H, Drula E, Coutinho PM, Henrissat B. 2014. The carbohydrate-active enzymes database (CAZy) in 2013. Nucleic Acids Res 42:D490–D495. doi:10.1093/nar/gkt1178 24270786 PMC3965031

[47] Rawlings ND, Barrett AJ, Finn R. 2016. Twenty years of the MEROPS database of proteolytic enzymes, their substrates and inhibitors. Nucleic Acids Res 44:D343–D350. doi:10.1093/nar/gkv1118 26527717 PMC4702814

[48] Altschul SF, Gish W, Miller W, Myers EW, Lipman DJ. 1990. Basic local alignment search tool. J Mol Biol 215:403–410. doi:10.1016/S0022-2836(05)80360-2 2231712

[49] Teufel F, Almagro Armenteros JJ, Johansen AR, Gíslason MH, Pihl SI, Tsirigos KD, Winther O, Brunak S, von Heijne G, Nielsen H. 2022. SignalP 6.0 predicts all five types of signal peptides using protein language models. Nat Biotechnol 40:1023–1025. doi:10.1038/s41587-021-01156-3 34980915 PMC9287161

[50] Yu NY, Wagner JR, Laird MR, Melli G, Rey S, Lo R, Dao P, Sahinalp SC, Ester M, Foster LJ, Brinkman FSL. 2010. PSORTb 3.0: improved protein subcellular localization prediction with refined localization subcategories and predictive capabilities for all prokaryotes. Bioinformatics 26:1608–1615. doi:10.1093/bioinformatics/btq249 20472543 PMC2887053

[51] Caspi R, Billington R, Ferrer L, Foerster H, Fulcher CA, Keseler IM, Kothari A, Krummenacker M, Latendresse M, Mueller LA, Ong Q, Paley S, Subhraveti P, Weaver DS, Karp PD. 2016. The MetaCyc database of metabolic pathways and enzymes and the BioCyc collection of pathway/genome databases. Nucleic Acids Res 44:D471–80. doi:10.1093/nar/gkv1164 26527732 PMC4702838

[52] von Meijenfeldt FAB, Arkhipova K, Cambuy DD, Coutinho FH, Dutilh BE. 2019. Robust taxonomic classification of uncharted microbial sequences and bins with CAT and BAT. Genome Biol 20:217. doi:10.1186/s13059-019-1817-x 31640809 PMC6805573

[53] Nguyen L-T, Schmidt HA, von Haeseler A, Minh BQ. 2015. IQ-TREE: a fast and effective stochastic algorithm for estimating maximum-likelihood phylogenies. Mol Biol Evol 32:268–274. doi:10.1093/molbev/msu300 25371430 PMC4271533

[54] Kalyaanamoorthy S, Minh BQ, Wong TKF, von Haeseler A, Jermiin LS. 2017. ModelFinder: fast model selection for accurate phylogenetic estimates. Nat Methods 14:587–589. doi:10.1038/nmeth.4285 28481363 PMC5453245

[55] Bateman A, Martin M-J, Orchard S, Magrane M, Agivetova R, Ahmad S, Alpi E, Bowler-Barnett EH, Britto R, Bursteinas B. 2021. UniProt: the universal protein knowledgebase in 2021. Nucleic Acids Res 49:D480–D489. doi:10.1093/nar/gkaa1100 33237286 PMC7778908

[56] Letunic I, Bork P. 2021. Interactive Tree Of Life (iTOL) v5: an online tool for phylogenetic tree display and annotation. Nucleic Acids Res 49:W293–W296. doi:10.1093/nar/gkab301 33885785 PMC8265157

[57] Zhu QY, Kosoy M, Dittmar K. 2014. HGTector: an automated method facilitating genome-wide discovery of putative horizontal gene transfers. BMC Genomics 15:717. doi:10.1186/1471-2164-15-717 25159222 PMC4155097

[58] Daims H, Lücker S, Wagner M. 2016. A new perspective on microbes formerly known as nitrite-oxidizing bacteria. Trends Microbiol 24:699–712. doi:10.1016/j.tim.2016.05.004 27283264 PMC6884419

[59] Liao TH, Wang SS, Stüeken EE, Luo HW. 2022. Phylogenomic evidence for the origin of obligate anaerobic anammox bacteria around the great oxidation event. Mol Biol Evol 39:msac170. doi:10.1093/molbev/msac170 35920138 PMC9387917

[60] Zhao R, Biddle JF, Jørgensen SL. 2022. Introducing Candidatus bathyanammoxibiaceae, a family of bacteria with the anammox potential present in both marine and terrestrial environments. ISME Commun 2:42. doi:10.1038/s43705-022-00125-4 37938673 PMC9723696

[61] Nunoura T, Nishizawa M, Kikuchi T, Tsubouchi T, Hirai M, Koide O, Miyazaki J, Hirayama H, Koba K, Takai K. 2013. Molecular biological and isotopic biogeochemical prognoses of the nitrification-driven dynamic microbial nitrogen cycle in hadopelagic sediments. Environ Microbiol 15:3087–3107. doi:10.1111/1462-2920.12152 23718903

[62] Walsh EA, Kirkpatrick JB, Rutherford SD, Smith DC, Sogin M, D’Hondt S. 2016. Bacterial diversity and community composition from seasurface to subseafloor. ISME J 10:979–989. doi:10.1038/ismej.2015.175 26430855 PMC4796937

[63] Kerou M, Ponce-Toledo RI, Zhao R, Abby SS, Hirai M, Nomaki H, Takaki Y, Nunoura T, Jørgensen SL, Schleper C. 2021. Genomes of Thaumarchaeota from deep sea sediments reveal specific adaptations of three independently evolved lineages. ISME J 15:2792–2808. doi:10.1038/s41396-021-00962-6 33795828 PMC8397731

[64] Pachiadaki MG, Sintes E, Bergauer K, Brown JM, Record NR, Swan BK, Mathyer ME, Hallam SJ, Lopez-Garcia P, Takaki Y, Nunoura T, Woyke T, Herndl GJ, Stepanauskas R. 2017. Major role of nitrite-oxidizing bacteria in dark ocean carbon fixation. Science 358:1046–1051. doi:10.1126/science.aan8260 29170234

[65] Kalvelage T, Lavik G, Lam P, Contreras S, Arteaga L, Löscher CR, Oschlies A, Paulmier A, Stramma L, Kuypers MMM. 2013. Nitrogen cycling driven by organic matter export in the South Pacific oxygen minimum zone. Nature Geosci 6:228–234. doi:10.1038/ngeo1739

[66] Spieck E, Lipski A. 2011. Cultivation, growth physiology, and chemotaxonomy of nitrite-oxidizing bacteria. Methods Enzymol 486:109–130. doi:10.1016/B978-0-12-381294-0.00005-5 21185433

[67] Sun X, Frey C, Garcia-Robledo E, Jayakumar A, Ward BB. 2021. Microbial niche differentiation explains nitrite oxidation in marine oxygen minimum zones. ISME J 15:1317–1329. doi:10.1038/s41396-020-00852-3 33408366 PMC8114937

[68] Yang N, Lv YX, Ji MK, Wu SG, Zhang Y. 2024. High hydrostatic pressure stimulates microbial nitrate reduction in hadal trench sediments under oxic conditions. Nat Commun 15:2473. doi:10.1038/s41467-024-46897-2 38503798 PMC10951307

[69] Palatinszky M, Herbold C, Jehmlich N, Pogoda M, Han P, von Bergen M, Lagkouvardos I, Karst SM, Galushko A, Koch H, Berry D, Daims H, Wagner M. 2015. Cyanate as an energy source for nitrifiers. Nature 524:105–108. doi:10.1038/nature14856 26222031 PMC4539577

[70] Alfreider A, Grimus V, Luger M, Ekblad A, Salcher MM, Summerer M. 2018. Autotrophic carbon fixation strategies used by nitrifying prokaryotes in freshwater lakes. FEMS Microbiol Ecol 94:fiy163. doi:10.1093/femsec/fiy163 30137292 PMC6118323

[71] Zhang Y, Qin W, Hou L, Zakem EJ, Wan XH, Zhao ZH, Liu L, Hunt KA, Jiao NZ, Kao SJ, Tang K, Xie XB, Shen JM, Li YF, Chen MM, Dai XF, Liu C, Deng WC, Dai MH, Ingalls AE, Stahl DA, Herndl GJ. 2020. Nitrifier adaptation to low energy flux controls inventory of reduced nitrogen in the dark ocean. Proc Natl Acad Sci U S A 117:4823–4830. doi:10.1073/pnas.1912367117 32071230 PMC7060736

[72] Wikström M, Krab K, Sharma V. 2018. Oxygen activation and energy conservation by cytochrome c oxidase. Chem Rev 118:2469–2490. doi:10.1021/acs.chemrev.7b00664 29350917 PMC6203177

[73] Arai H, Kawakami T, Osamura T, Hirai T, Sakai Y, Ishii M. 2014. Enzymatic characterization and in vivo function of five terminal oxidases in Pseudomonas aeruginosa. J Bacteriol 196:4206–4215. doi:10.1128/JB.02176-14 25182500 PMC4248849

[74] Kawakami T, Kuroki M, Ishii M, Igarashi Y, Arai H. 2010. Differential expression of multiple terminal oxidases for aerobic respiration in Pseudomonas aeruginosa. Environ Microbiol 12:1399–1412. doi:10.1111/j.1462-2920.2009.02109.x 19930444

[75] Le Laz S, Kpebe A, Bauzan M, Lignon S, Rousset M, Brugna M. 2016. Expression of terminal oxidases under nutrient-starved conditions in Shewanella oneidensis: detection of the A-type cytochrome c oxidase. Sci Rep 6:19726. doi:10.1038/srep19726 26815910 PMC4728554

[76] Okabe S, Shafdar AA, Kobayashi K, Zhang L, Oshiki M. 2021. Glycogen metabolism of the anammox bacterium “Candidatus Brocadia sinica”. ISME J 15:1287–1301. doi:10.1038/s41396-020-00850-5 33288860 PMC8115630

[77] Wolfe AJ. 2015. Glycolysis for microbiome generatio. Microbiol Spectr 3. doi:10.1128/microbiolspec.MBP-0014-2014 PMC4507297 26185089

[78] Oshiki M, Satoh H, Okabe S. 2016. Ecology and physiology of anaerobic ammonium oxidizing bacteria. Environ Microbiol 18:2784–2796. doi:10.1111/1462-2920.13134 26616750

[79] Yang Y, Lu Z, Azari M, Kartal B, Du H, Cai M, Herbold CW, Ding X, Denecke M, Li X, Li M, Gu JD. 2022. Discovery of a new genus of anaerobic ammonium oxidizing bacteria with a mechanism for oxygen tolerance. Water Research 226:119165. doi:10.1016/j.watres.2022.119165 36257158

[80] Glud RN, Berg P, Thamdrup B, Larsen M, Stewart HA, Jamieson AJ, Glud A, Oguri K, Sanei H, Rowden AA, Wenzhöfer F. 2021. Hadal trenches are dynamic hotspots for early diagenesis in the deep sea. Commun Earth Environ 2:21. doi:10.1038/s43247-020-00087-2

[81] Kuhn T, Versteegh GJM, Villinger H, Dohrmann I, Heller C, Koschinsky A, Kaul N, Ritter S, Wegorzewski AV, Kasten S. 2017. Widespread seawater circulation in 18–22 Ma oceanic crust: impact on heat flow and sediment geochemistry. Geology 45:799–802. doi:10.1130/G39091.1

[82] Rastelli E, Corinaldesi C, Dell’Anno A, Tangherlini M, Lo Martire M, Nishizawa M, Nomaki H, Nunoura T, Danovaro R. 2019. Drivers of bacterial alpha- and beta-diversity patterns and functioning in subsurface hadal sediments. Front Microbiol 10:2609. doi:10.3389/fmicb.2019.02609 31798555 PMC6868121

[83] Kraft B, Jehmlich N, Larsen M, Bristow LA, Könneke M, Thamdrup B, Canfield DE. 2022. Oxygen and nitrogen production by an ammonia-oxidizing archaeon. Science 375:97–100. doi:10.1126/science.abe6733 34990242

[84] Lam P, Kuypers MMM. 2011. Microbial nitrogen cycling processes in oxygen minimum zones. Ann Rev Mar Sci 3:317–345. doi:10.1146/annurev-marine-120709-142814 21329208

[85] Hutchins DA, Capone DG. 2022. The marine nitrogen cycle: new developments and global change. Nat Rev Microbiol 20:401–414. doi:10.1038/s41579-022-00687-z 35132241

[86] Harwood CS. 2020. Iron-only and vanadium nitrogenases: fail-safe enzymes or something more?. Annu Rev Microbiol 74:247–266. doi:10.1146/annurev-micro-022620-014338 32660386

[87] Zhang XN, Ward BB, Sigman DM. 2020. Global nitrogen cycle: critical enzymes, organisms, and processes for nitrogen budgets and dynamics. Chem. Rev 120:5308–5351. doi:10.1021/acs.chemrev.9b00613 32530264

[88] Chen YX, Nishihara A, Haruta S. 2021. Nitrogen-fixing ability and nitrogen fixation-related genes of thermophilic fermentative bacteria in the genus Caldicellulosiruptor. Microbes Environ 36:ME21018. doi:10.1264/jsme2.ME21018 34108360 PMC8209448

[89] Dong XY, Zhang CW, Peng YY, Zhang HX, Shi LD, Wei GS, Hubert CRJ, Wang Y, Greening C. 2022. Phylogenetically and catabolically diverse diazotrophs reside in deep-sea cold seep sediments. Nat Commun 13:4885. doi:10.1038/s41467-022-32503-w 35985998 PMC9391474

[90] Bullock HA, Shen H, Boynton TO, Shimkets LJ. 2018. Fatty acid oxidation is required for Myxococcus xanthus development. J Bacteriol 200:e00572-17. doi:10.1128/JB.00572-17 29507089 PMC5915784

[91] Kim SH, Kim JG, Jung MY, Kim SJ, Gwak JH, Yu WJ, Roh SW, Kim YH, Rhee SK. 2018. Ketobacter alkanivorans gen. nov., sp nov., an n-alkane-degrading bacterium isolated from seawater. Int J Syst Evol Microbiol 68:2258–2264. doi:10.1099/ijsem.0.002823 29809120

[92] Yakimov MM, Golyshin PN, Crisafi F, Denaro R, Giuliano L. 2019. Marine, aerobic hydrocarbon-degrading *Gammaproteobacteria*: the family *Alcanivoracaceae*, p 1–13. In McGenity T (ed), Taxonomy, genomics and ecophysiology of hydrocarbon-degrading microbes, Handbook of Hydrocarbon and Lipid Microbiology. Springer, Cham.

[93] Liu Y, Chen SZ, Xie Z, Zhang L, Wang JH, Fang JS. 2023. Influence of extremely high pressure and oxygen on hydrocarbon-enriched microbial communities in sediments from the Challenger Deep, Mariana Trench. Microorganisms 11:630. doi:10.3390/microorganisms11030630 36985204 PMC10052102

[94] Li WL, Huang JM, Zhang PW, Cui GJ, Wei ZF, Wu YZ, Gao ZM, Han Z, Wang Y. 2019. Periodic and spatial spreading of alkanes and Alcanivorax bacteria in deep waters of the Mariana Trench. Appl Environ Microbiol 85:e02089-18. doi:10.1128/AEM.02089-18 30446553 PMC6344633

[95] Xu Y, Li X, Luo M, Xiao W, Fang J, Rashid H, Peng Y, Li W, Wenzhöfer F, Rowden AA, Glud RN. 2021. Distribution, source, and burial of sedimentary organic carbon in Kermadec and Atacama Trenches. JGR Biogeosciences 126:e2020JG006189. doi:10.1029/2020JG006189

