# Supplementary material for: Distinct microbial nitrogen cycling processes in the deepest part of the ocean
Source: mSystems. 2024 Jun 28;9(7):e00243-24. doi: 10.1128/msystems.00243-24 (PMC11265455; doi:10.1128/msystems.00243-24)
Supplement: Supplemental Figures — Figures S1 to S10. [file msystems.00243-24-s0001.pdf]

Supplementary figures for
Distinct microbial nitrogen cycling processes in the deepest part of the ocean

Yuhan Huang,[a,b] Xinxu Zhang,[a,b] Yu Xin,[c] Jiwei Tian,[d] Meng Li[a,b,#]

[a]Archaeal Biology Center, Shenzhen Key Laboratory of Marine Microbiome Engineering, Institute for Advanced Study, Shenzhen University, Shenzhen 518060, PR China
[b]Synthetic Biology Research Center, Institute for Advanced Study, Shenzhen University, Shenzhen 518060, PR China
[c]Key Laboratory of Marine Chemistry Theory and Technology, Ministry of Education, Institute for Advanced Ocean Study, Ocean University of China, Qingdao, Shandong, China
[d]MOE Key Laboratory of Physical Oceanography, Frontiers Science Center for Deep Ocean Multispheres and Earth System, Ocean University of China, Qingdao, China

#Address correspondence to Meng Li, limeng848@szu.edu.cn (Meng Li)
Yuhan Huang and Xinxu Zhang contributed equally to this work. Author order was determined by drawing straws.

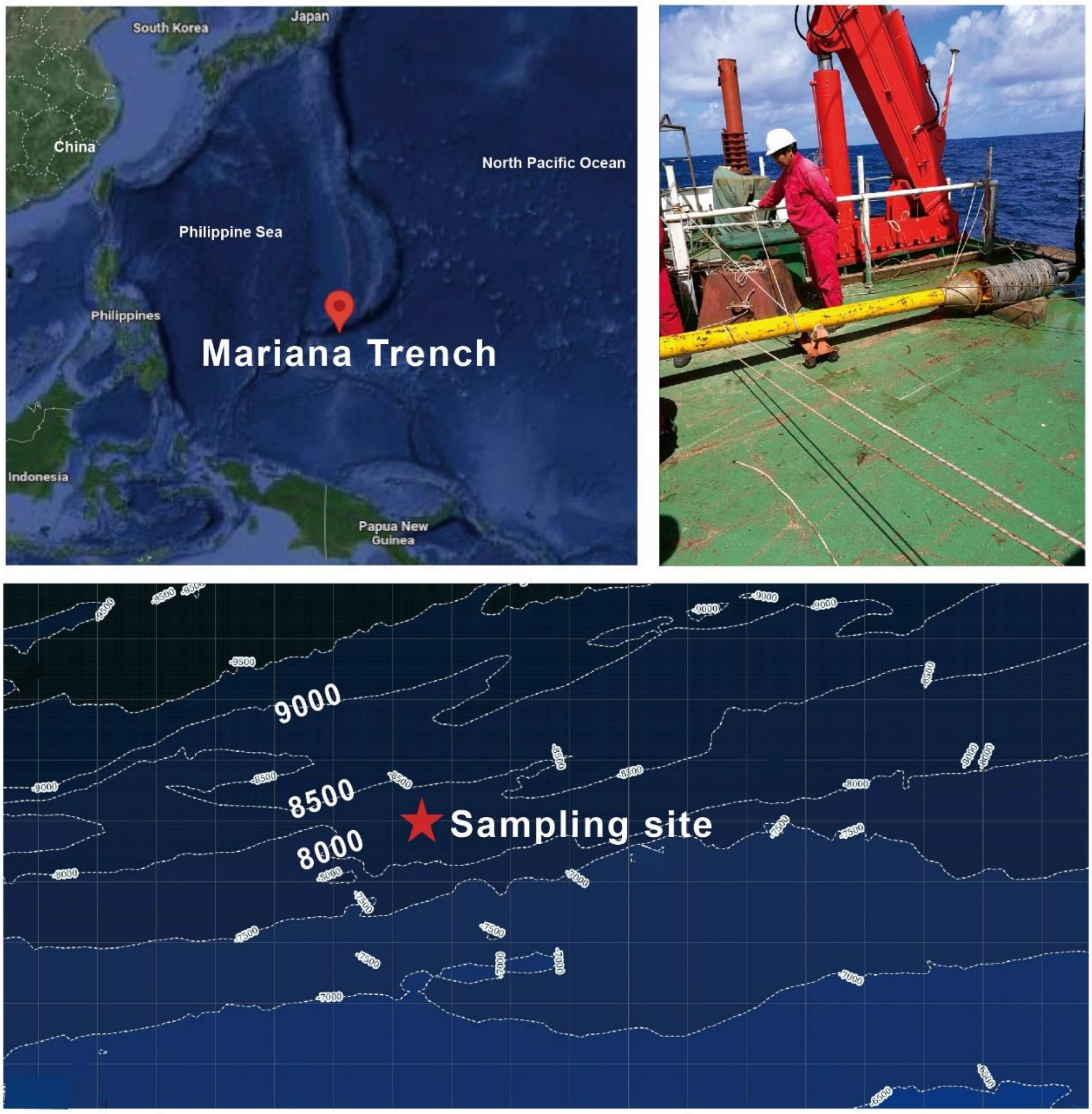

Figure S1. Geographic location of the sampling site, the Mariana Trench.

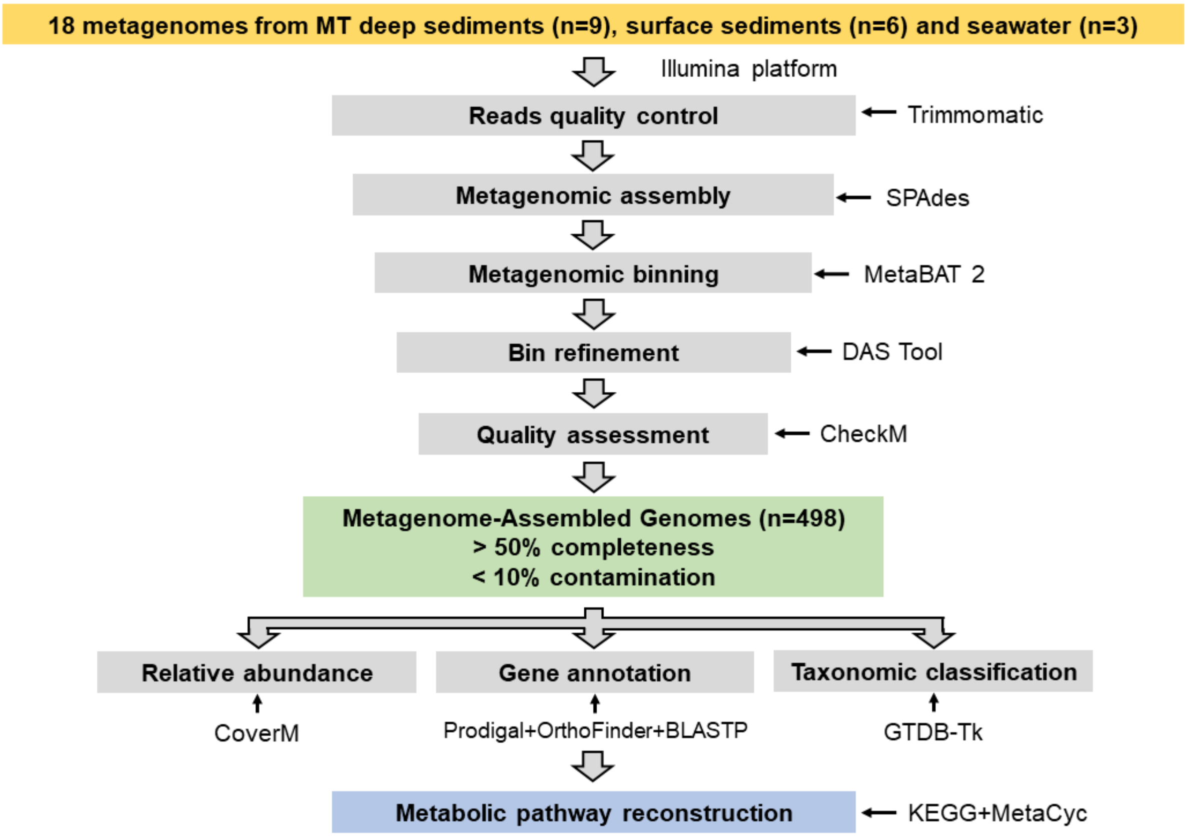

Figure S2. A detailed workflow of the metagenomic analysis used in this study.

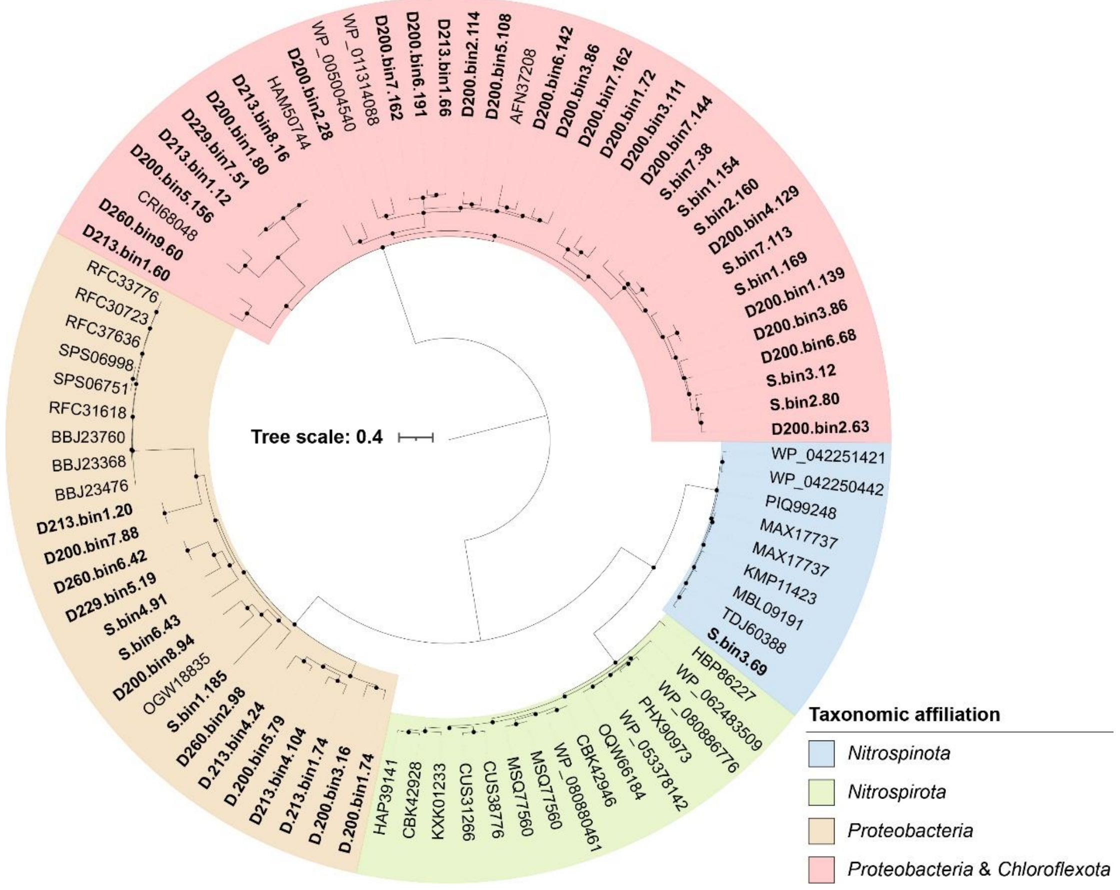

Figure S3. Phylogenetic tree of the nitrite oxidoreductase gene *nxrA*. Different colors covering the tree branches indicate the taxonomic affiliation of the *nxrA* genes. The sequences from this study are indicated in bold font. Ultrafast bootstrapping is used to estimate the reliability of each branch with 1,000 times resampling, and the nodes with a bootstrap value >70 are marked with black dots.

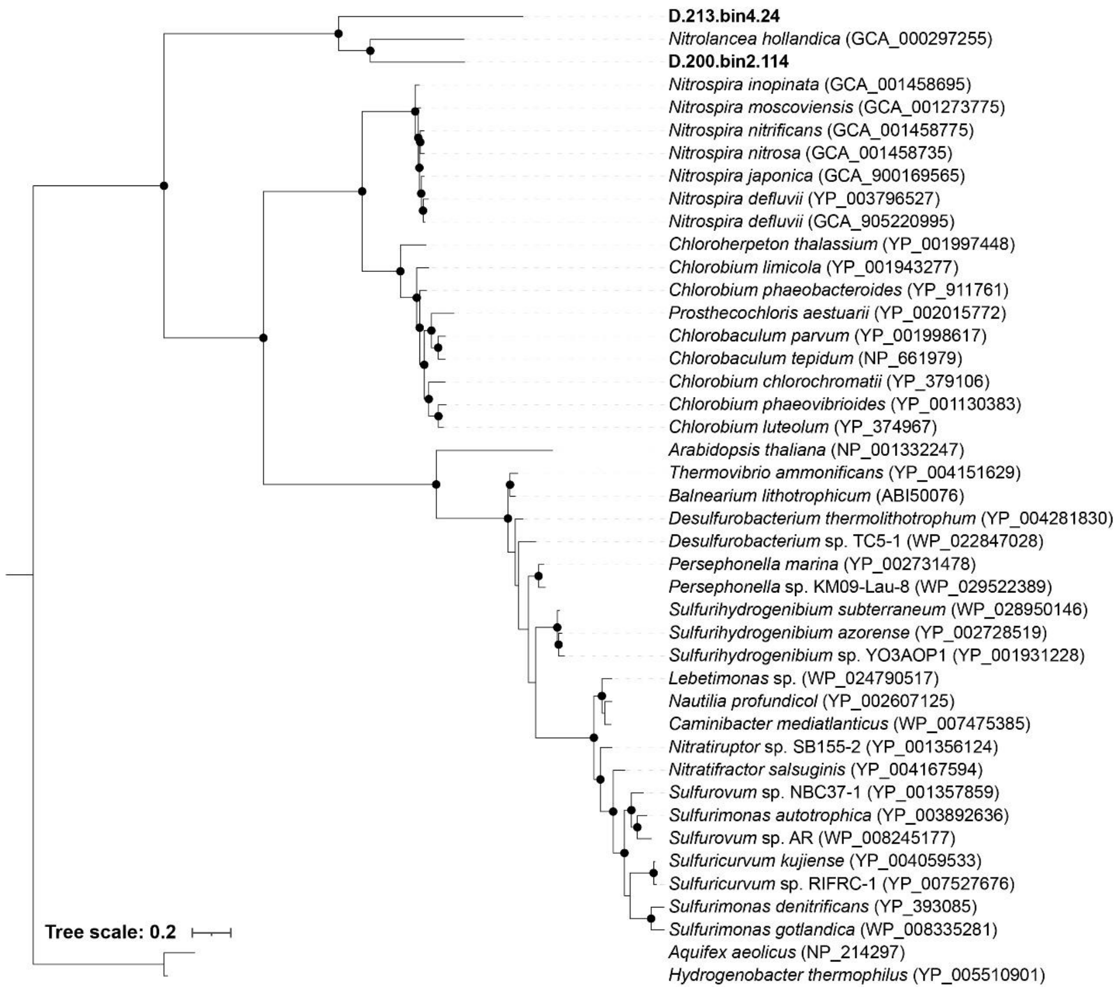

Figure S4. Phylogenetic tree of the ATP citrate lyase alpha subunit gene *aclA*. The succinyl-CoA ligase alpha subunit gene is used as the outgroup. The sequences from this study are indicated in bold font. Ultrafast bootstrapping is used to estimate the reliability of each branch with 1,000 times resampling, and the nodes with a bootstrap value >70 are marked with black dots.

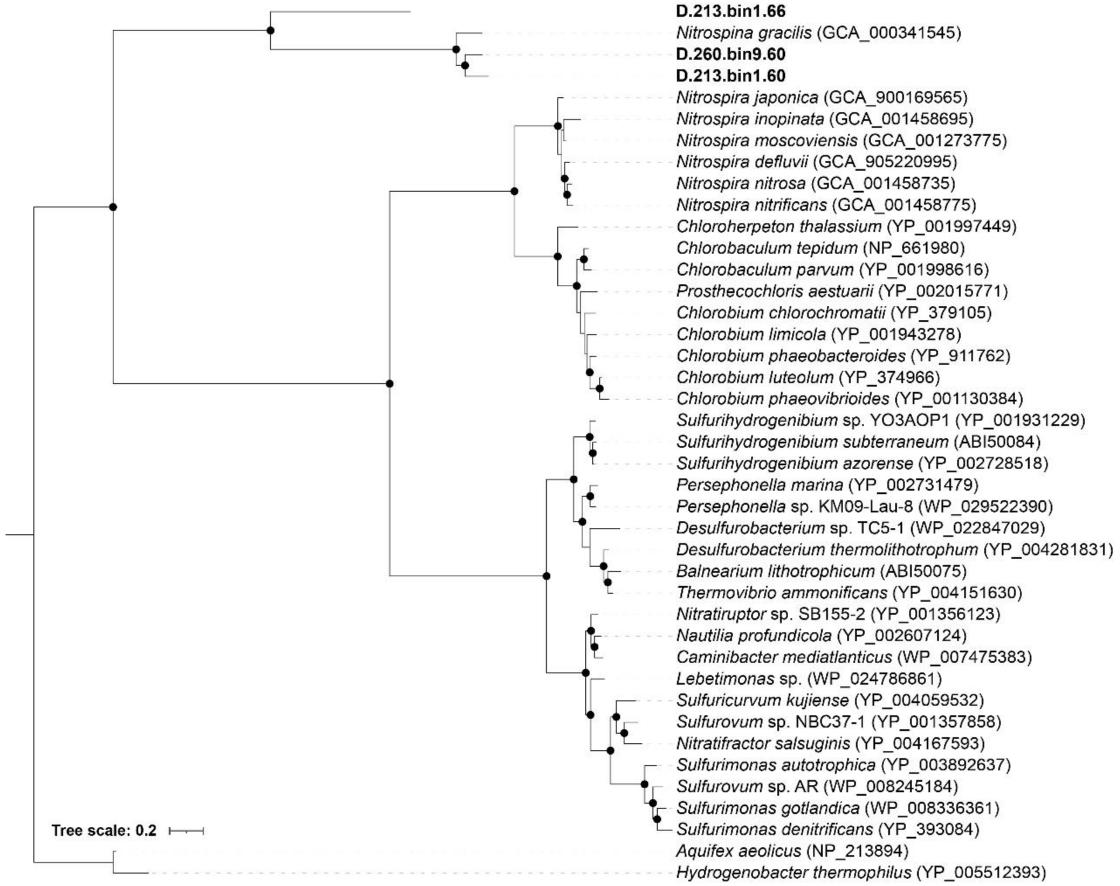

Figure S5. Phylogenetic tree of the ATP citrate lyase beta subunit gene *aclB*. The succinyl-CoA ligase beta subunit gene is used as the outgroup. The sequences from this study are indicated in bold font. Ultrafast bootstrapping is used to estimate the reliability of each branch with 1,000 times resampling, and the nodes with a bootstrap value >70 are marked with black dots.

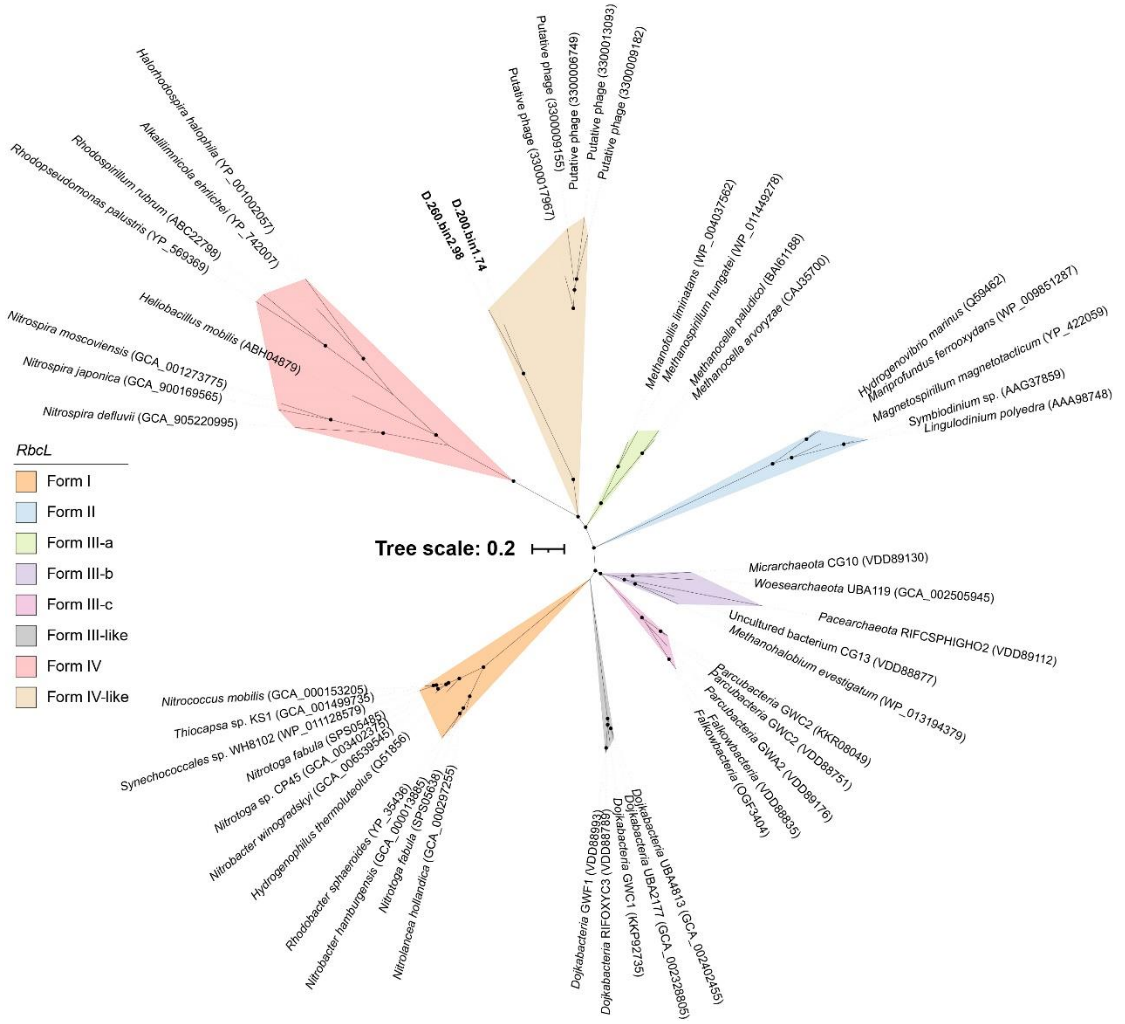

Figure S6. Phylogenetic tree of the ribulose-bisphosphate carboxylase large subunit gene *rbcL*. Different colors covering the tree branches indicate the eight *rbcL* gene forms, as described elsewhere [1]. The sequences from this study are indicated in bold font. Ultrafast bootstrapping is used to estimate the reliability of each branch with 1,000 times resampling, and the nodes with a bootstrap value >70 are marked with black dots.

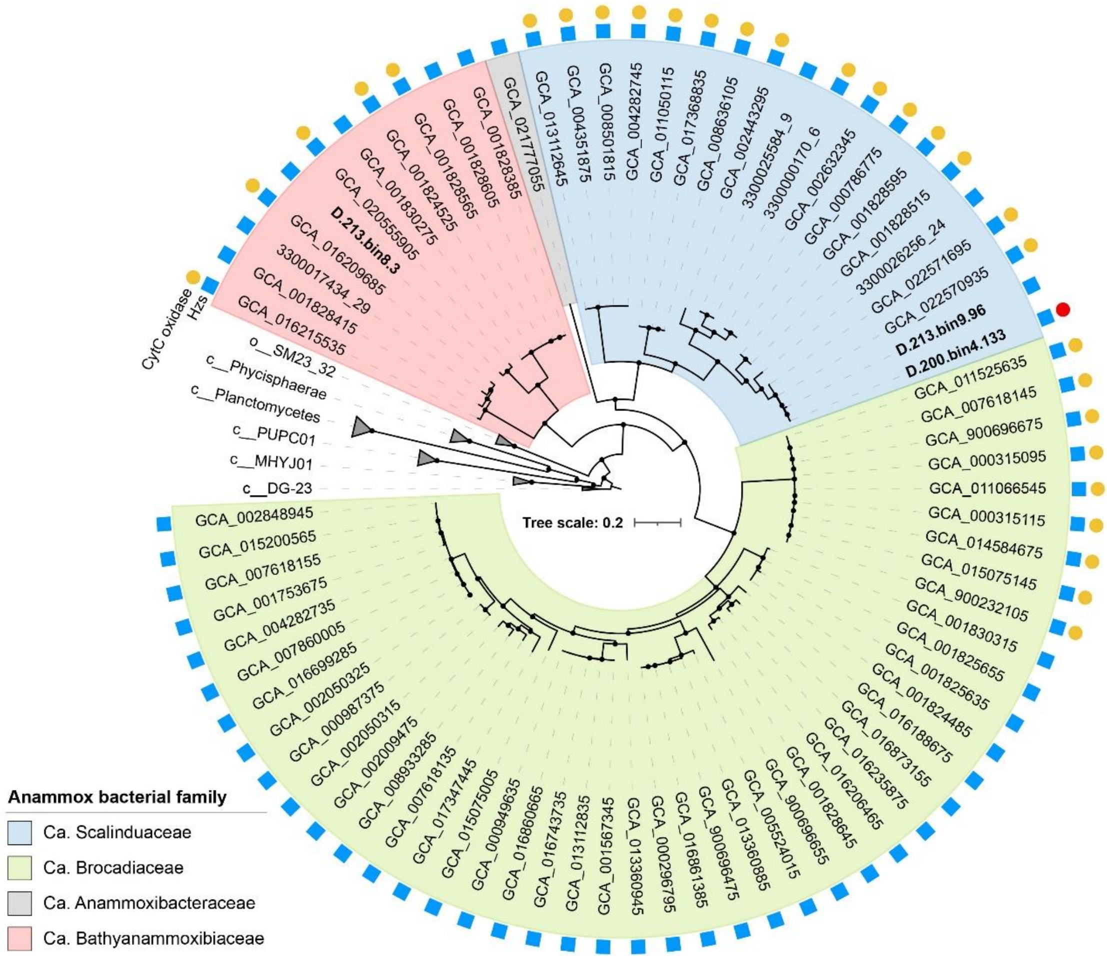

Figure S7. Phylogenomic tree of the anammox bacterial genomes. Different colors covering the tree branches indicate the four anammox bacteria families. Each filled circle or square next to the genome ID indicates the presence of the cytochrome *c* (CytC) oxidase or hydrazine synthase (*hzs*) gene in the MAG, respectively. The red and yellow filled circle indicate the type A and type C cytochrome *c* oxidase genes, respectively. The MAGs from this study are indicated in bold font. Ultrafast bootstrapping is used to estimate the reliability of each branch with 1,000 times resampling, and the nodes with a bootstrap value >80 are marked with black dots.

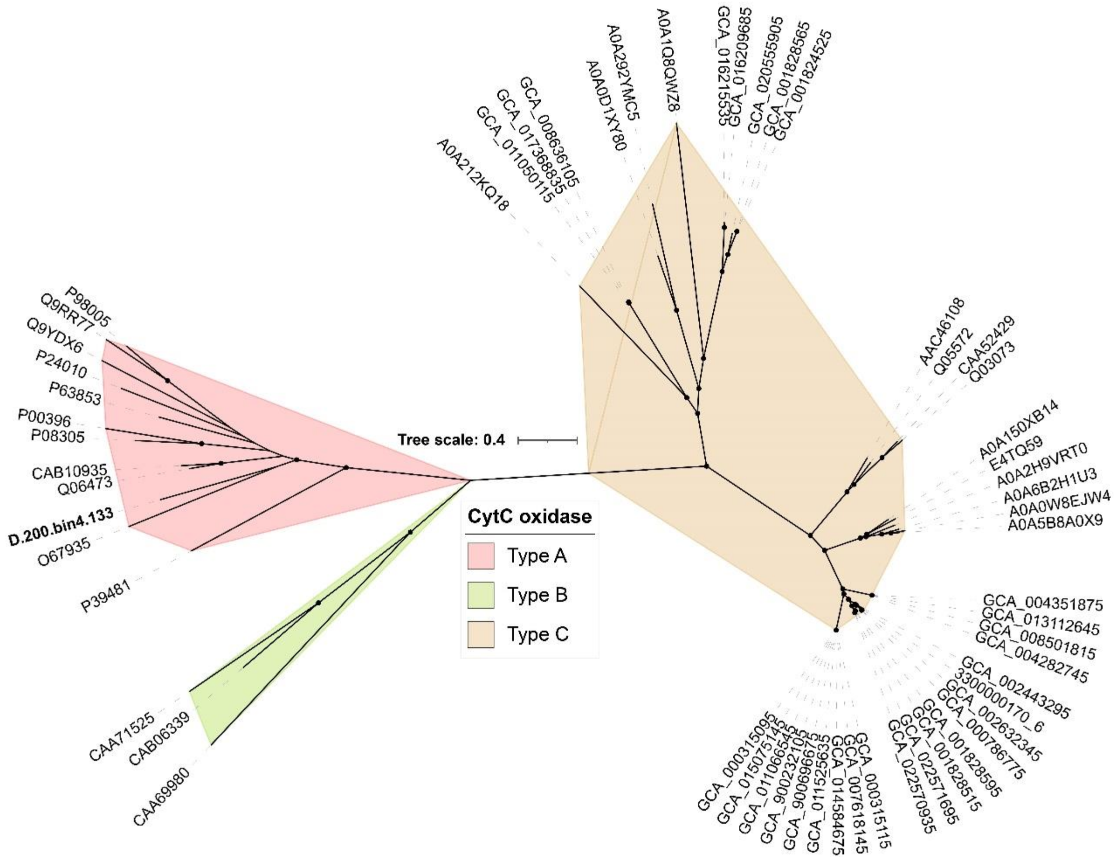

Figure S8. Phylogenetic tree of the cytochrome *c* oxidase gene. Different colors covering the tree branches indicate the three CytC oxidase gene types. The sequence from this study is indicated in bold font. Ultrafast bootstrapping is used to estimate the reliability of each branch with 1,000 times resampling, and the nodes with a bootstrap value >70 are marked with black dots.

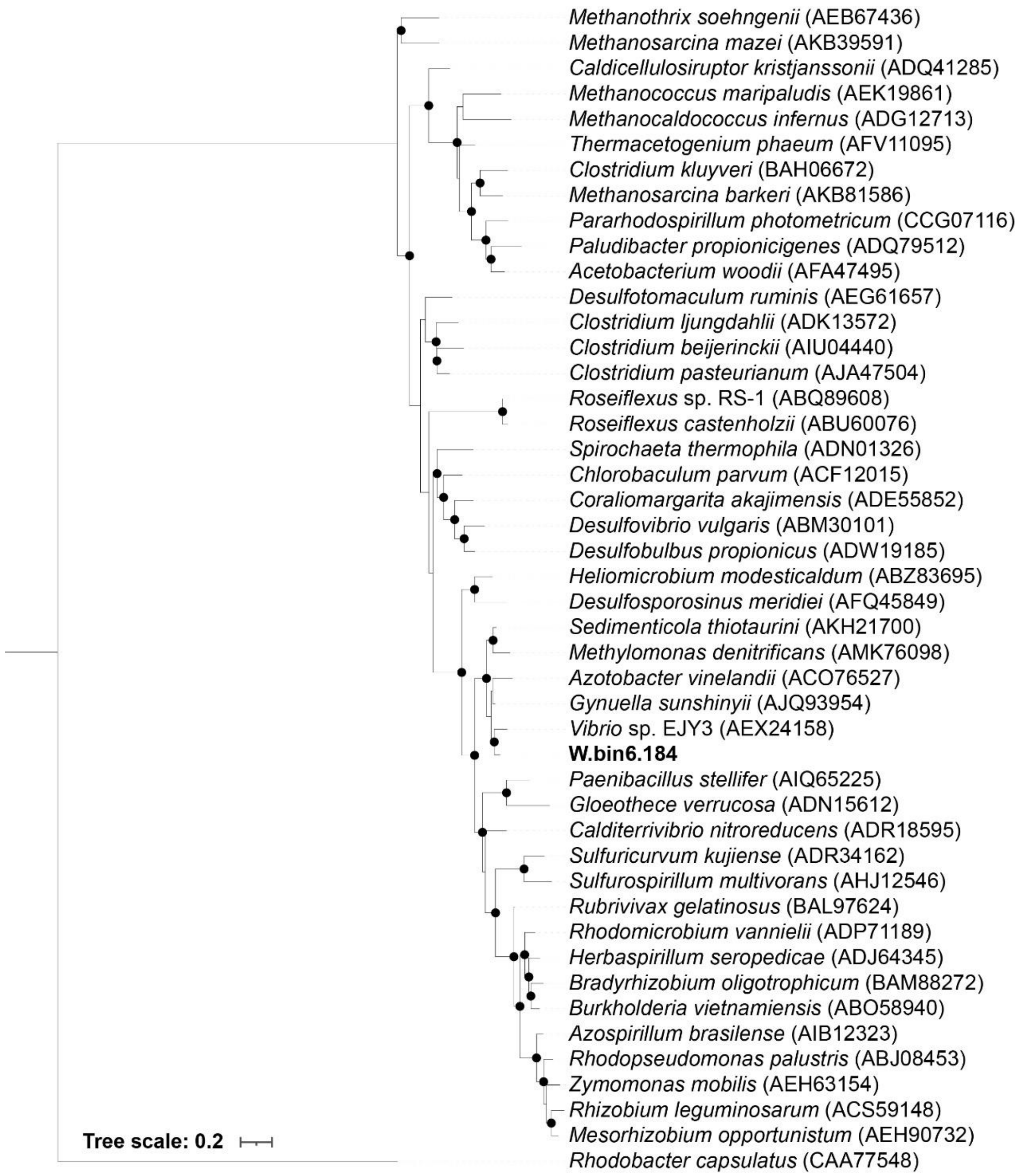

Figure S9. Phylogenetic tree of the nitrogenase gene *nifH*. The chlorophyllide reductase gene *bchX* is used as the outgroup. The sequences from this study are indicated in bold font. Ultrafast bootstrapping is used to estimate the reliability of each branch with 1,000 times resampling, and the nodes with a bootstrap value >70 are marked with black dots.

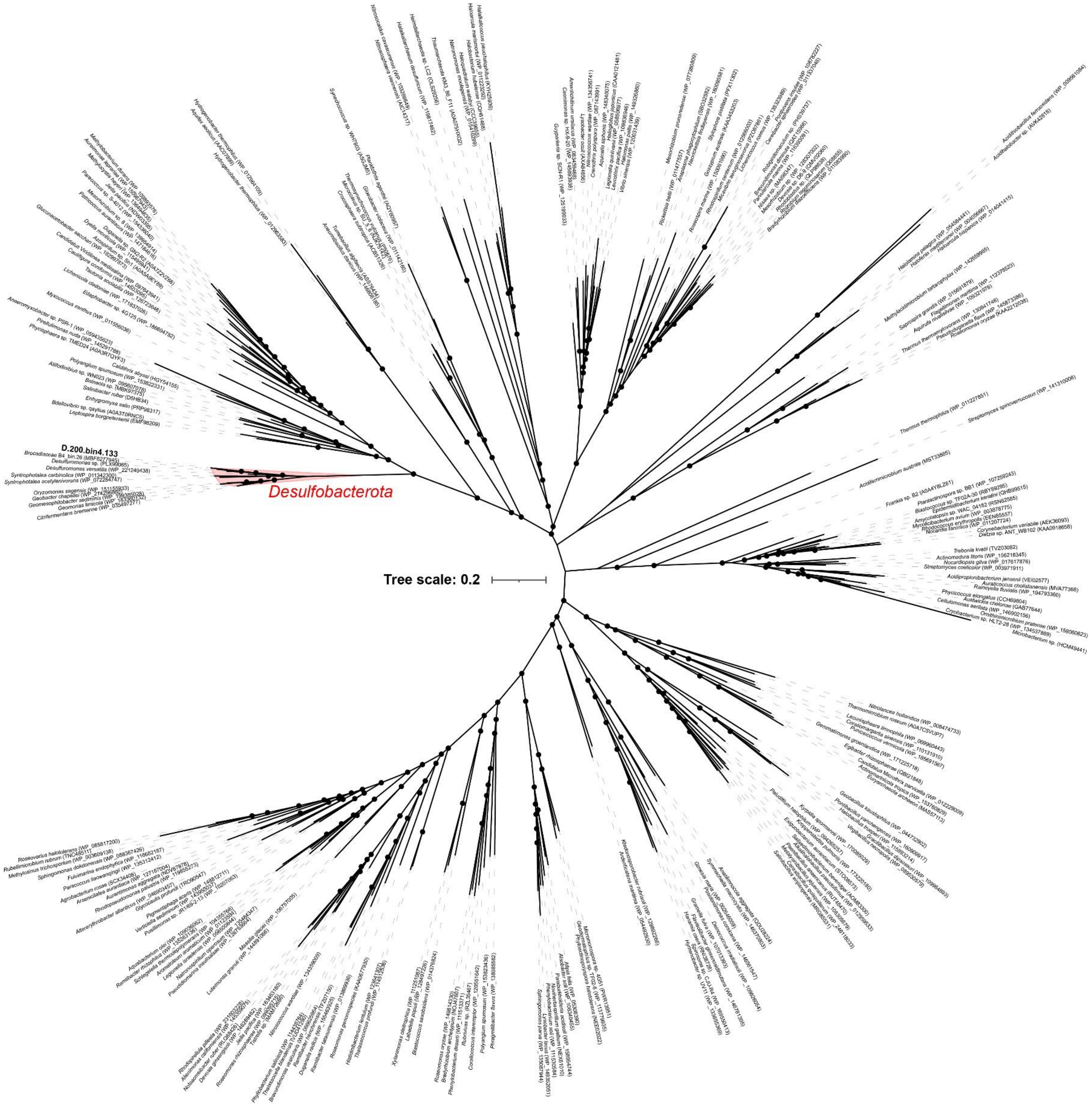
D.200.bin4.133
Desulfobacterota
Tree scale: 0.2

Figure S10. Phylogenetic tree showing the origin of the type A cytochrome *c* oxidase gene in anammox bacterium (D.200.bin4.133). The sequence from this study is indicated in bold font. The tree branches covered with red color indicate *Desulfobacterota*-derived genes. Ultrafast bootstrapping is used to estimate the reliability of each branch with 1,000 times resampling, and the nodes with a bootstrap value >70 are marked with black dots.

Reference

1. Pan J, Zhou ZC, Béjà O, Cai MW, Yang YC, Liu Y, Gu JD, Li M. 2020. Genomic and transcriptomic evidence of light-sensing, porphyrin biosynthesis, Calvin-Benson-Bassham cycle, and urea production in *Bathyarchaeota*. Microbiome 8:43. doi.org/10.1186/s40168-020-00820-1.
